# Didehydro-Cortistatin A Inhibits HIV-1 by Specifically Binding to the Unstructured Basic Region of Tat

**DOI:** 10.1128/mBio.02662-18

**Published:** 2019-02-05

**Authors:** Sonia Mediouni, Krishna Chinthalapudi, Mary K. Ekka, Ippei Usui, Joseph A. Jablonski, Mark A. Clementz, Guillaume Mousseau, Jason Nowak, Venkat R. Macherla, Jacob N. Beverage, Eduardo Esquenazi, Phil Baran, Ian Mitchelle S. de Vera, Douglas Kojetin, Erwann P. Loret, Kendall Nettles, Souvik Maiti, Tina Izard, Susana T. Valente

**Affiliations:** a Department of Immunology and Microbiology, The Scripps Research Institute, Jupiter, Florida, USA; b CSIR-Institute of Genomics and Integrative Biology, New Delhi, India; c Department of Integrative Structural and Computational Biology, The Scripps Research Institute, Jupiter, Florida, USA; d Sirenas, La Jolla, California, USA; e Department of Chemistry, The Scripps Research Institute, La Jolla, California, USA; f Aix Marseille University, University of Avignon, IMBE UMR CNRS 7263, IRD 237, Faculty of Pharmacy, Avignon, France; g Department of Pharmacology and Physiology, St. Louis University School of Medicine, St. Louis, Missouri, USA; QIMR Berghofer Medical Research Institute; Albert Einstein College of Medicine

**Keywords:** HIV-1 Tat, structure-activity relationship, dCA, molecular docking, structure-based design

## Abstract

The intrinsically disordered HIV-1 Tat protein binds the viral RNA transactivation response structure (TAR), which recruits transcriptional cofactors, amplifying viral mRNA expression. Limited Tat transactivation correlates with HIV-1 latency. Unfortunately, Tat inhibitors are not clinically available. The small molecule didehydro-cortistatin A (dCA) inhibits Tat, locking HIV-1 in persistent latency, blocking viral rebound. We generated chemical derivatives of dCA that rationalized molecular docking of dCA to an active and specific Tat conformer. These revealed the importance of the cycloheptene ring and the isoquinoline nitrogen’s positioning in the interaction with specific residues of Tat’s basic domain. These features are distinct from the ones required for inhibition of cyclin-dependent kinase 8 (CDK8), the only other known ligand of dCA. Besides, we demonstrated that dCA activity on HIV-1 transcription is independent of CDK8. The binding of dCA to Tat with nanomolar affinity alters the local protein environment, rendering Tat more resistant to proteolytic digestion. dCA thus locks a transient conformer of Tat, specifically blocking functions dependent of its basic domain, namely the Tat-TAR interaction; while proteins with similar basic patches are unaffected by dCA. Our results improve our knowledge of the mode of action of dCA and support structure-based design strategies targeting Tat, to help advance development of dCA, as well as novel Tat inhibitors.

## Abstract (precis)

Tat activates virus production, and limited Tat transactivation correlates with HIV-1 latency. The Tat inhibitor dCA locks HIV in persistent latency. This drug class enables block-and-lock functional cure approaches, aimed at reducing residual viremia during therapy and limiting viral rebound. dCA may also have additional therapeutic benefits since Tat is also neurotoxic. Unfortunately, Tat inhibitors are not clinically available. We generated chemical derivatives and rationalized binding to an active and specific Tat conformer. dCA features required for Tat inhibition are distinct from features needed for inhibition of cyclin-dependent kinase 8 (CDK8), the only other known target of dCA. Furthermore, knockdown of CDK8 did not impact dCA’s activity on HIV-1 transcription. Binding of dCA to Tat’s basic domain altered the local protein environment and rendered Tat more resistant to proteolytic digestion. dCA locks a transient conformer of Tat, blocking functions dependent on its basic domain, namely its ability to amplify viral transcription. Our results define dCA’s mode of action, support structure-based-design strategies targeting Tat, and provide valuable information for drug development around the dCA pharmacophore.

## INTRODUCTION

The HIV-1 transactivator of transcription, Tat, is a prototypical intrinsically disordered protein, which enables its multiple functions, with variable folding dependent on the binding partner, (1, 2). Tat is expressed in the early stages of infection and plays a crucial role in amplifying HIV expression and maintaining residual viremia during suppressive antiretroviral therapy (ART) (3). Tat binds to the 5′-terminal region of HIV mRNA’s stem-bulge-loop structure, the transactivation response (TAR) element, through a disordered basic patch located in the center of the protein. The N-terminal activation domain of Tat recruits the positive transcription elongation factor, P-TEFb (composed of cyclin T1 and cyclin-dependent kinase 9 [CDK9]), to TAR, resulting in exponential amplification of viral mRNA production (4). Tat is also a secreted protein that crosses membranes and the blood-brain barrier, causing neurotoxicity (5). This membrane-crossing property is a function of the basic patch and is so robust that the Tat basic peptide has been used as a general tool for protein delivery into cells (6).

Didehydro-cortistatin A (dCA) is an analog of the natural product cortistatin A (CA). CA has been reported to display antiproliferative properties in human umbilical vein endothelial cells (HUVECs) (50% effective concentration [EC_50_], 0.35 µM) and antileukemic activity in CA-sensitive AML cell lines (50% inhibitory concentration [IC_50_] ∼10 nM) by selectively inhibiting CDK8 activity (7 – 9). We have shown that dCA potently inhibits Tat-mediated HIV transcription (IC_50_, ∼1 to 2 nM) (10, 11). dCA inhibits HIV-1 from primary CD4^+^ T cells isolated from infected ART-suppressed individuals and blocks viral rebound upon treatment interruption (12). dCA also inhibits extracellular uptake of Tat by glial cells (13), thus inhibiting both intracellular and secreted Tat activities. Using deletion mutants of full-length Tat and individual Tat peptides, we identified Tat’s basic patch to be fundamental for the interaction with dCA (10, 13). Over time, inhibition of Tat by long-term treatment with dCA results in epigenetic modifications, locking the HIV-1 promoter in persistent latency. We observed increased histone occupancy at nucleosome 1, restricting RNA polymerase II (RNAPII) recruitment to the HIV-1 promoter. The *in vivo* efficacy of dCA was demonstrated in the bone marrow-liver-thymus mouse model of HIV latency and persistence, where addition of dCA to ART-suppressed mice systemically reduced viral mRNA in tissues and significantly delayed and reduced viral rebound levels upon treatment interruption (12).

The structural context of Tat's basic domain has been studied using five available NMR structures of Tat proteins present in the Protein Data Bank (PDB), which contain the N-terminus (residues 1 to 48) and basic patch (residues 49 to 59) (Table 1). These revealed ensembles of a motional structure, where the basic patch laid across the surface and displayed no secondary structure, while the side chains were exposed to the solvent. NMR structures determined with active Tat protein reveal that several of the arginine residues in this basic region form H-bonds with the N-terminus, suggesting the basic patch stabilizes this conformer (Table 1) (2, 14, 15). These intramolecular interactions were found in several Tat variants (14, 16), with the residue Asp^2^/Glu^2^ directly interacting with Lys^51^ and Arg^53^ of the basic domain, and mutation of residue 2 resulting in destabilization and loss of activity. Asp^2^/Glu^2^ correlates motion with other Tat domains and provides the largest electrostatic stabilization of the protein. Furthermore, Yezid and colleagues (15) demonstrated that residues Asp^2^/Glu^2^ along with basic domain residues (residues 55 to 57) form a pH sensor controlling Tat structure. Other studies however, in which the bioactivity of Tat was not demonstrated, show the activation domain fully or partially disordered (Table 1).

**TABLE 1:** Characteristics of Tat structures present in the Protein Data Bank^*a*^

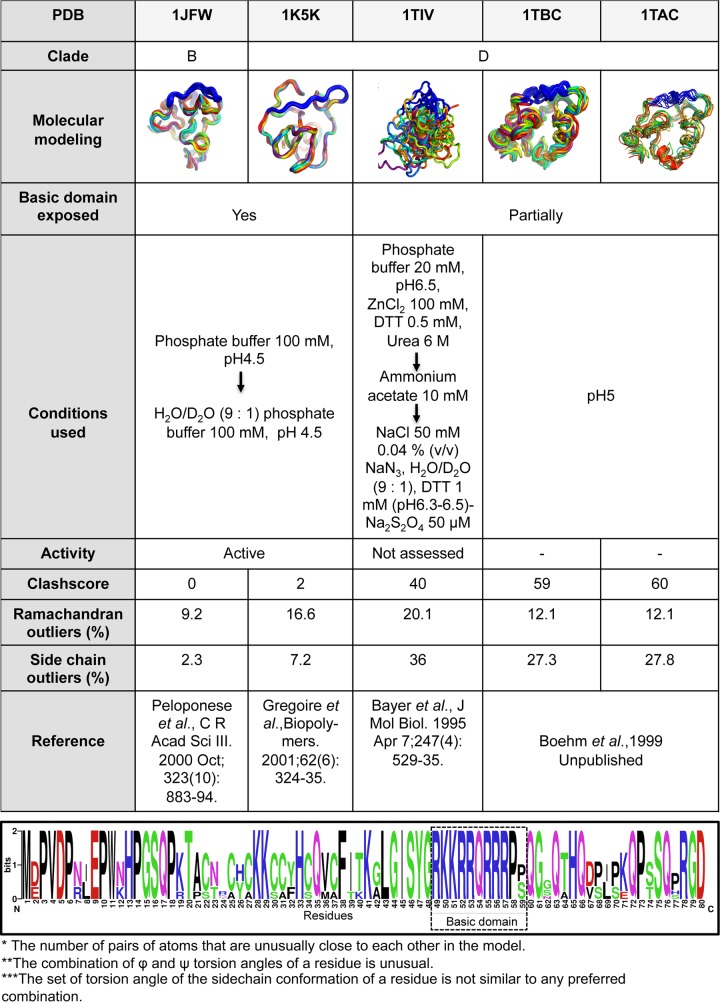	

a (Top) Table summarizing the conditions and structural details of the 5 molecular models of Tat present in the Protein Data Bank containing both exons of the protein. (Bottom) Weblogo of the first 80 residues of Tat from NMR structures present in PDB 1K5K, 1JFW, 1TAC, 1TIV, and 1TBC (http://weblogo.berkeley.edu/logo.cgi). Letter size is proportional to residue conservation.

Tat’s structure is sensitive to pH, oxidation, and binding to cellular partners, such as membrane-bound phosphatidylinositol 4,5-bisphosphate [PI(4,5)P_2_] or P-TEFb (17 – 19). Upon binding to P-TEFb, Tat adopts a highly extended conformer in which residues 1 to 48 fold around the cyclin T1 subunit and also contact the T loop of the CDK9 subunit, while the remainder of the protein, including the basic domain, is disordered (see Fig. S1 in the supplemental material). In this conformer, the cysteines are brought together to coordinate a zinc with cyclin T1 at the interface (18, 19). It is not known how dCA binds to the basic patch with respect to these different conformers.

10.1128/mBio.02662-18.2 FIG S1 Crystal structure of HIV-1 Tat complexed with ATP-bound human P-TEFb. Residues 1 to 48 of Tat protein are resolved in the structure with visible α-helices for the C-terminus region (residues 28 to 43). Despite being unstructured, the N terminus of Tat protein is stabilized by the P-TEFb protein in the complex. Cyclin T1 is colored in dark green, CDK9 in gray, and Tat in purple. This figure was prepared using data from PDB entry 3MIA. Download FIG S1, PDF file, 2 MB. Copyright © 2019 Mediouni et al. 2019 Mediouni et al. This content is distributed under the terms of the Creative Commons Attribution 4.0 International license.

Here, we characterize the molecular interaction of Tat with dCA. We show by isothermal titration calorimetry (ITC) assay that dCA binds with nanomolar affinity to Tat. Structure-function relationship studies of dCA and analogs, combined with molecular modeling using NMR structures of Tat (20), revealed dCA binding to Tat in a stable conformer rather than an unstructured one. These studies revealed the importance of the nitrogen atom in the isoquinoline moiety of dCA, as well as the cycloheptene ring in the interaction with the basic region (Lys^51^, Arg^52^, and Arg^55^) and N-terminal region (Pro^3^) of Tat. These features are distinct from the ones required for CDK8 inhibition, the only other known ligand of dCA. In addition, the knockdown of CDK8, or its paralog, CDK19, did not alter HIV-1 transcription or the anti-HIV activity of dCA. Proteolysis protection assays, tryptophan fluorescence, and cell-based protection assays revealed a stabilization of Tat structure by dCA. We excluded an activity of dCA on proteins with similar basic patches such as Hexim-1 (hexamethylene bisacetamide inducible 1) and HIV-1 Rev. dCA disruption of the interaction of the Tat basic domain with TAR was demonstrated by electrophoretic mobility shift assays (EMSA) and chromatin immunoprecipitation (ChIP) studies. Together our results improve our knowledge of the mechanism of action of dCA and help rationalize the development of the dCA pharmacophore as an antiviral drug.

## RESULTS

### dCA directly interacts with Tat.

**FIG 1:**
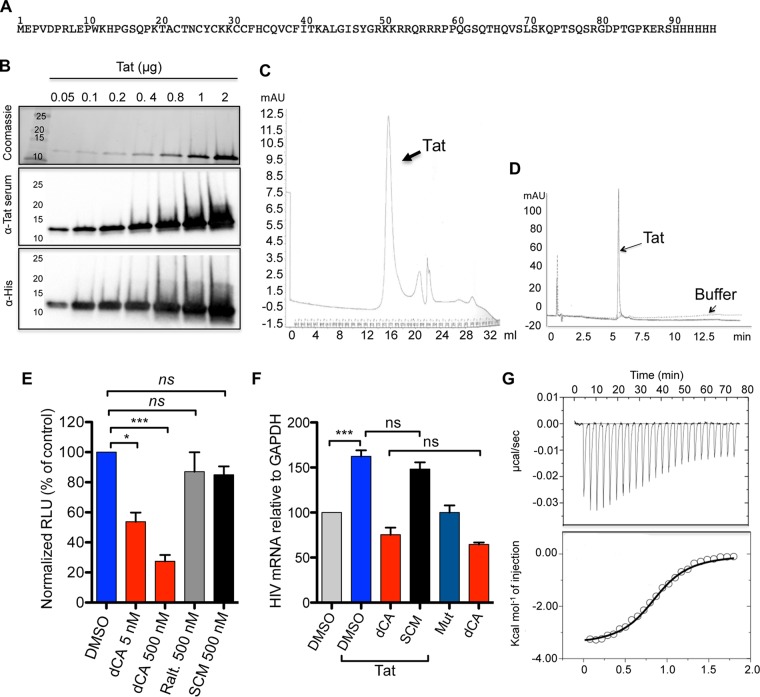
Purity and activity of the monomeric His-tagged Tat HXB3 and analysis of its direct binding to dCA. (A) Amino acid sequence of His-tagged-Tat HXB3 protein. (B) Analysis of recombinant Tat purity and monomeric status upon Coomassie staining and Western blotting with an anti-Tat serum or anti-His antibody. Data are representative of *n* = 3 independent experiments. (C) Size exclusion chromatography of the recombinant Tat. (D) HPLC of the recombinant Tat. Data in panels C and D are representative of *n* = 2 independent experiments. (E) dCA inhibits the transactivation activity of recombinant Tat in HeLa-CD4-LTR-Luc cells. Raltegravir (Ralt.) and compound *S*-(−)-carbidopamonohydrate (SCM) were used as negative controls. Data are the mean ± standard error of the mean (SEM) from *n* = 3 independent experiments. (F) dCA inhibits the transactivation activity of recombinant Tat in the OM10.1 cell model of HIV-1 latency. Compound SCM and transactivation with recombinant Tat Mut were used as negative controls. Data are the mean ± standard deviation (SD) from *n* = 3 independent experiments. (G) ITC of dCA binding to Tat. The top plot shows the baseline corrected experimental data. In the bottom plot, results were converted to molar heat and plotted against the molar ratio of dCA to Tat protein. Data are representative of *n* = 2 independent experiments. Statistical significance was determined using one-way ANOVA with *post hoc* Tukey’s test. ns, not significant; ***, *P* < 0.0001; *, *P* < 0.01.

The production of functional recombinant HIV-1 Tat in Escherichia coli is not trivial as the proper folding of Tat is extremely important for activity, and when expressed by itself, Tat agglomerates mostly because of oxidation of the cysteine residues in the cysteine region. Tat can also adhere to different surfaces and anionic polymers *via* its charged and hydrophobic residues (21, 22). We produced and purified recombinant HIV Tat HXB3 (residues 1 to 89) with a 6×His tag in the C-terminus in E. coli. For this, we have partially optimized the arginine codon usage and changed the rare AGA into codon CGT at arginine positions 7, 53, 56, and 87 of Tat (Fig. 1A). These changes in combination with the use of M9 medium resulted in the production of approximately 5 mg/liter of purified Tat. After Tat purification using nickel affinity chromatography followed by size exclusion filtration, Tat protein size was analyzed by SDS-PAGE followed by Coomassie blue staining (Fig. 1B). The specificity of the bands was confirmed by Western blotting using anti-Tat serum and anti-His antibody (Fig. 1B). The purity of Tat was further verified by size exclusion chromatography and high-performance liquid chromatography (HPLC) (Fig. 1C and D). We confirmed the activity of Tat by performing transactivation assays in both HeLa-CD4 cells stably expressing the HIV-1 5′ long terminal repeat (LTR) promoter driving luciferase expression (HeLa-CD4-LTR-Luc cells) (10) and the OM10.1 cell line model of HIV-1 latency (23) (Fig. 1E and F; see Fig. S2A and S2B in the supplemental material). In HeLa-CD4-LTR-Luc cells, the activity of our purified Tat was similar to the published activity of Tat obtained from the AIDS Reagent Program (10). In addition, denatured Tat and Tat mutated in the basic domain (Tat Mut [arginine residues mutated to alanine residues]), used as negative controls, were unable to transactivate the reporter (Fig. S2A). As expected, dCA blocked Tat transcriptional activity in a dose-dependent manner (Fig. 1E). Controls such as raltegravir, an HIV-1 integrase inhibitor, and SCM [*S*-(−)-carbidopamonohydrate], a nonspecific compound (which inhibits l-amino acid decarboxylase) not known to block HIV replication, had no inhibitory activity. In OM10.1 cells, Tat significantly activated HIV-1 transcription, while Tat Mut did not (Fig. 1F). The addition of dCA (250 nM) inhibited transcriptional activity of Tat (1 μg) to the same extent as dCA alone (Fig. 1F), while SCM, used as a negative control, had no activity.

10.1128/mBio.02662-18.3 FIG S2 Tat transactivation activity. Shown is transactivation activity in (A) HeLa-CD4-LTR-Luc cells and (B) OM10.1 cells. Relative light units (RLU) show luciferase per total protein. Data are the mean ± SD from *n* = 3 independent experiments. Statistical significance was determined using one-way ANOVA with *post hoc* Tukey’s test. ns, not significant; ***, *P* < 0.0001; **, *P* < 0.001. Download FIG S2, PDF file, 0.1 MB. Copyright © 2019 Mediouni et al. 2019 Mediouni et al. This content is distributed under the terms of the Creative Commons Attribution 4.0 International license.

Next, we studied the direct interaction of Tat with dCA using ITC. After gradual titration of Tat with dCA in the ITC cell, the integrated heat data of the dCA-Tat interaction were collected (Fig. 1G). This ITC titration curve fitted well with a single-site binding process with a nonlinear least-squares regression, allowing the determination of the association constant (*K_a_*) and entropy (Δ*S*) and enthalpy (Δ*H*) changes, as well as the average number of binding sites (*N*). We determined a 1:1 binding stoichiometry with a *K_a_* of ∼(9.39 ± 1.7) × 10^6^ M^−1^ (dissociation constant [*K_d_*] = 100 nM). Moreover, dCA binds to Tat at 25°C with a negative (favorable) enthalpy of −3.5 ± 0.1 kcal mol^−1^ and positive (favorable) entropy of +20.0 ± 0.5 cal mol^−1^ K^−1^. The favorable values of Δ*H* and Δ*S* are consistent with the characteristics of a combination of van der Waals, hydrophobic, and electrostatic interactions in the binding process. The difference between Tat/dCA affinity (ITC, 100 nM) and dCA inhibitory activity in cell-based assays (0.6 to 2 nM) (Table 2) (10) could be the result of the nature of the Tat-TAR feedback loop that amplifies activity *in vivo*; especially since the amount of Tat *in vivo* after infection is very low (24).

**TABLE 2:** Structure-activity relationship of dCA^*a*^

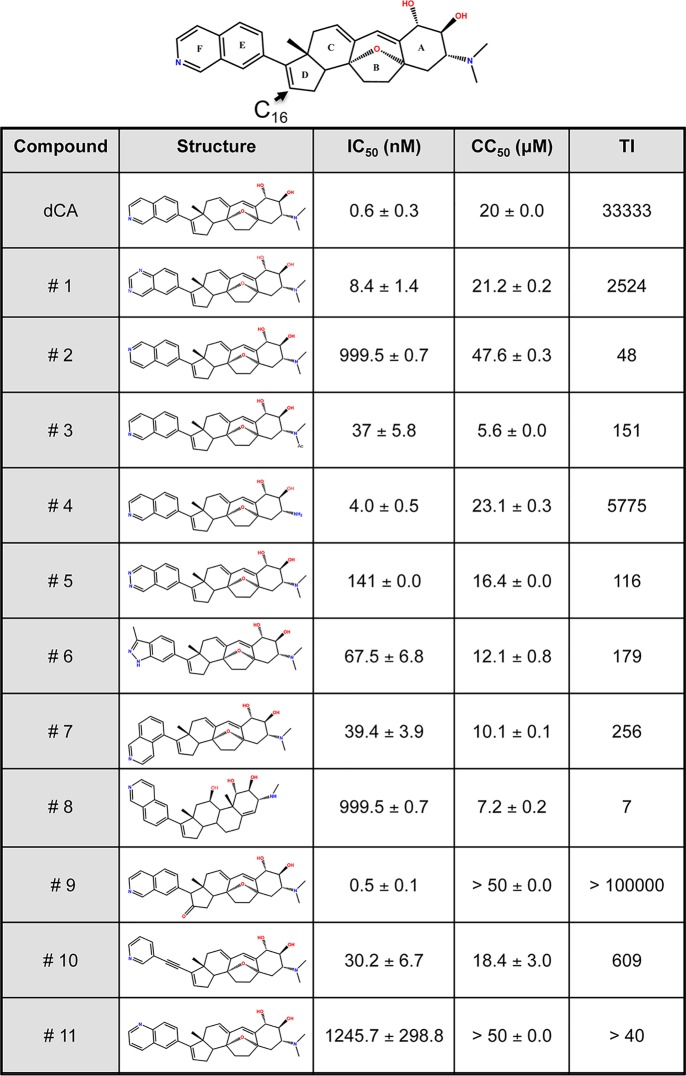

a IC_50_, half-maximal inhibitory concentration. HeLa-CD4-LTR-LacZ cells were infected with the NL4-3 strain in the presence of increasing concentrations of each dCA analog. LacZ was measured 72 h later. CC_50_, half-maximal cytotoxic concentration. HeLa-CD4-LTR-LacZ cells were incubated with each analog for 72 h, followed by an MTT viability assay. Results are the mean ± SD from *n* = 2 to 3 independent experiments. TI, therapeutic index.

### Blind modeling of dCA binding to Tat’s active conformation of the activation and basic domains.

**FIG 2:**
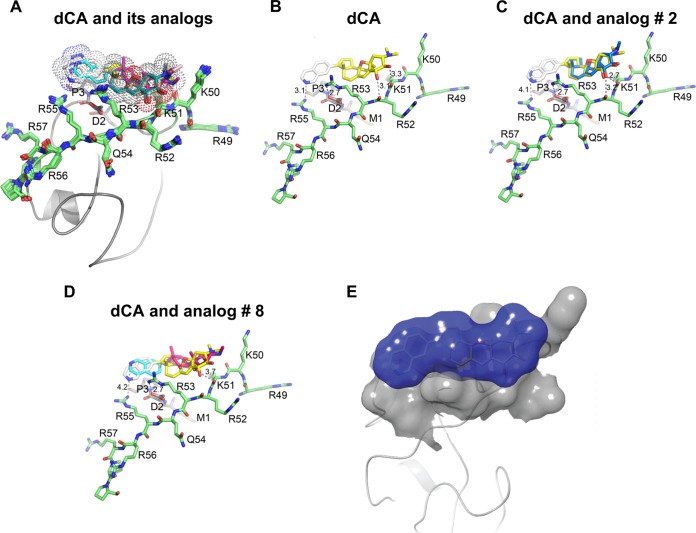
Molecular modeling of the interaction of dCA with HIV-1 Tat protein PDB entry 1K5K. (A) The small molecule dCA and analogs with similar scaffold preferentially docked to the basic patch of the HIV-1 Tat protein. (B) Close-up view of the best pose of dCA binding to Tat in the docking analysis. Basic patch residues of the NMR ensemble are shown in stick representation, and the ligand dCA is shown in yellow. (C and D) Close-up view of the binding site of two inactive analogs of dCA (analogs 2 and 8) and the interacting residues in HIV-1 Tat protein. (E) Overview of dCA binding to Tat protein—a horseshoe-shaped dCA binding site. The basic patch of Tat is shown as a gray surface, and dCA compound is shown in stick representation with a transparent blue surface.

We previously showed that dCA inhibits Tat from HIV clades A, B, C, D, and E (13); thus, molecular docking of dCA to Tat was performed using AutoDock Vina and the HIV-1 Tat Mal protein (D clade) as a template (PDB entry 1K5K) (Table 1 and Fig. 2). PDB entry 1K5K was chosen based on the following: (i) all its NMR ensembles are converged and energetically minimized, (ii) its structure and sequence superimpose with those of other Tat proteins (Table 1, Fig. 2; see Fig. S3 in the supplemental material), (iii) the basic patch residues are docked against the activation domain and are solvent exposed (Table 1 and Fig. 2), (iv) its structure was validated by biochemical assays and by mutagenesis (14, 15, 17, 25 – 29), (v) its NMR was performed under the active/functional conditions of the protein, and (vi) it has a low clashscore and low percentages of Ramachandran and side-chain outliers (Table 1). Initially, blind docking with dCA and similar scaffolds were done with a cubic grid size of 45 Å in AutoDock Vina, the exhaustiveness level was set at 10, and default options were used for the remaining parameters. Consistent binding poses were obtained, and the top 10 best-docked ligand poses were selected based on the free energy predictions (from −7.9 to −6.5 kcal/mol) (Fig. 2A). In most cases, the ligands were oriented in similar orientations, and those poses that were in opposite orientation differed in the free energies by <0.2 kcal/mol. We obtained the basic patch as the binding interface as the top two hits by blind docking. We further targeted the basic domain binding site for clear orientation of the ligands by reducing the grid size to 28 Å, approximately half the size of the blind docking grid. We obtained consistent docking sites with the orientation of dCA and other analogs in the basic patch (residues 49 to 59) with small changes in the free energies (Δ*G* of less than −0.1 kcal/mol). Only the top solution out of the 10 best hits of dCA was used for pharmacophore analysis of the Tat binding site. Our docking analysis showed that the small molecule dCA binds preferentially to the basic patch of Tat protein. On one side of the dCA molecule, basic patch residue Arg^55^ directly interacts with its nitrogen atom (N^27^) on the isoquinoline group (Fig. 2B). Furthermore, we performed similar docking experiments with additional Tat structure PDB entries 1JFW, 1TIV and 1TBC (1TAC was not included given its similarities to 1TBC). We found the binding of dCA to Tat to be similar, with the isoquinoline and intact cycloheptene groups dictating dCA’s ability to bind to the basic patch of the HIV-1 Tat proteins (see Fig. S3 and S4 in the supplemental material).

10.1128/mBio.02662-18.4 FIG S3 Molecular modeling of interaction of Tat PDB entry 1JFW with dCA. (A) The small molecule dCA and other analogs with a similar scaffold preferentially docked to the basic patch of HIV-1 Tat protein PDB entry 1JFW. (B) Close-up view of the best pose of dCA molecule binding to Tat in the docking analysis. Basic patch residues of the NMR ensemble are shown in stick representation, and the ligand dCA is shown in yellow. (C and D) Close-up view of the binding site of two inactive analogs of dCA (analogs 2 and 8) and the interacting residues in HIV-1 Tat protein. Download FIG S3, PDF file, 5 MB. Copyright © 2019 Mediouni et al. 2019 Mediouni et al. This content is distributed under the terms of the Creative Commons Attribution 4.0 International license.

10.1128/mBio.02662-18.5 FIG S4 Molecular modeling of Tat PDB entry 1TIV and 1TBC binding to dCA. (A) dCA preferentially docked to the basic patch of HIV-1 Tat protein in model 1 of the structure PBD entry 1TIV. Of note, residue Arg^55^, important for dCA binding to Tat, is buried under the C terminus of the Tat protein. (B) Ensemble of the conformations of the basic domain of PBD entry 1TIV, from residues Ile^45^ (I45) to Pro^58^ (P58), shown in stick representation. (C) Analysis of docking results using the PBD entry 1TBC model as a template showed similar docking orientations as PBD entry 1K5K model. Of note, some of the basic residues are buried in this structure and the Arg^53^ guanidinium group is in close proximity to tryptophan indole ring, which is energetically not favorable. Basic patch residues of the NMR ensemble are shown in stick representation, and the ligand dCA is shown in yellow. Docking analysis of other inactive analogs, analogs 2 (in blue) and 8 (in pink), are also shown for PBD entry 1TIV and 1TBC models. All docking experiments were performed as for the PBD entry 1K5K template. (D) Ensemble of the conformations of the basic domain of HIV-1 Tat in PBD entry 1TBC model, from residues Ile^45^ (I45) to Pro^58^ (P58), shown in stick representation. Download FIG S4, PDF file, 4 MB. Copyright © 2019 Mediouni et al. 2019 Mediouni et al. This content is distributed under the terms of the Creative Commons Attribution 4.0 International license.

### Structure-function relationship of dCA.

To understand the structure-function relationship of the dCA molecule with respect to our structural model, we synthesized and characterized the ability of 11 analogs of dCA to inhibit HIV infection (Table 2). Figure S5 in the supplemental material shows their NMR profiles. We used HeLa-CD4 cells stably expressing the HIV-1 5′ LTR driving β-galactosidase production (HeLa-CD4-LTR-LacZ cells) as a reporter for the infection with isolate NL4-3. Table 2 summarizes the IC_50_, 50% cytotoxic concentration (CC_50_), and therapeutic index (TI) for each compound. The loss of dCA activity was directly associated with the positioning and the chemical environment of the azote on the F ring and the presence of oxygen in ring B. Moreover, the E-F “skeleton angle” and the distance between the A-B-C-D and F rings affected the activity by more than 50-fold (analogs 7 and 10). Interestingly, the oxidation at position 16 in ring D improved the IC_50_ (from 0.6 nM to 0.5 nM for dCA versus analog 9), as well as the CC_50_ (from 20 μM to >50 μM) and the TI (from 33,333 to >100,000).

10.1128/mBio.02662-18.6 FIG S5 NMR profiles of dCA and analogs. Download FIG S5, PDF file, 0.4 MB. Copyright © 2019 Mediouni et al. 2019 Mediouni et al. This content is distributed under the terms of the Creative Commons Attribution 4.0 International license.

As we mentioned above, dCA is an analog of the natural product CA. CA has been reported to display antiproliferative properties toward HUVECs and antileukemic activity in CA-sensitive AML cell lines by selectively inhibiting CDK8 activity (7 – 9). Importantly, the characteristics necessary for the antiretroviral activity of dCA do not seem to overlap features needed for HUVECs’ antiangiogenic activity, which requires at least one OH or NMe_2_ in ring A, a certain A-B-C skeleton angle, no oxidation of C_16_ and C_17_ in ring D, and an intact stereochemistry of ring E-F (30).

In our structure-function relationship analysis, dCA and analogs 1, 3, 4, 8, and 9 (all active except 8) have the same orientation and position of nitrogen atom, and they are within hydrogen bond distance with the guanidinium group of Arg^55^ (Fig. 2A, B, and D). Importantly, all these molecules show higher affinities than the molecules with nitrogen atoms in different positions (Table 2). On the other side of dCA molecule, the backbone atoms of the basic residues Lys^51^ and Arg^52^ interact with the hydroxyl groups on the estrone group and stabilize the dCA molecule (Fig. 2B). However, even when the nitrogen atom of the analogs is in an optimal orientation and the cycloheptene group on the cortistatin scaffold is perturbed—for example, analog 8 (Fig. 2D)—these interactions are not stabilized as the hydroxyl groups are not interacting with the backbone atoms of the basic patch. In our structure-function relationship analysis, though the phthalazine derivative (analog 5) has a similar orientation to dCA and has one of the nitrogens in the same position as isoquinoline, it lacks the C-H group and is replaced by a nitrogen atom, reducing its affinity to Tat. The isoquinoline group of dCA readily forms a hydrogen bond with Arg^55^, and the adjacent C-H group readily forms a hydrogen bond with backbone carbonyl of Pro^3^ from the N-terminal region of Tat protein (see Fig. S6 in the supplemental material). Hence, we propose that the position and orientation of this nitrogen atom in the isoquinoline group and the adjacent C-H group are important. The heterocyclic C-H bond donors are typically stronger when the carbon atom carrying the donor is adjacent to a heteroatom in the ring, with electron-withdrawing elements or substituents amplifying the effect. Thus, we suggest that an isoquinoline group and an intact cycloheptene ring in these analogs are important for their potent inhibitor activity (Fig. 2B to D). These compounds are also stabilized by the hydrophobic noncharged carbon side chain atoms (-CH_2_-) tails of the basic patch residues Lys^50^, Lys^51^, and Arg^53^. Moreover, the N-terminal residue Glu^2^ interacts with Arg^53^, and the residue Pro^3^ interacts with the isoquinoline group, further stabilizing the binding surface for the dCA molecule. In sum, Lys^51^ and Arg^52^ interact with the hydroxyl groups of dCA, and the isoquinoline group of dCA forms a hydrogen bond with Arg^55^ and the backbone carbonyl of Pro^3^. The interaction of Glu^2^ with Arg^53^ may further stabilize the binding of dCA to Tat by bringing both domains (N-terminal and basic domains) into close proximity. Concurrently, the addition of dCA to the strong intramolecular interactions between both domains locks the protein in a more stable structure, further connecting both domains of the protein. All these residues in the basic patch form a horseshoe-like surface (Fig. 2E) for the bulky dCA ligand and similar scaffolds and enable their potent inhibitor properties. We observed similar binding of dCA and analogs to Tat using other known NMR structures (1JFW, 1TIV and 1TBC), and essentially the binding features to the basic patch residues are preserved (Fig. S3 and S4).

10.1128/mBio.02662-18.7 FIG S6 Structure-function activity of dCA and analog 5. (A) dCA contains an isoquinoline heterocyclic group, the nitrogen atom of this group interacts with the -NH_2_ moiety from the guanidinium group of Arg^55^, and the C-H group adjacent is in hydrogen bonding distance from the backbone carbonyl of the Pro^3^ residue from the N terminus of Tat. (B) Analog 5 contains a phthalazine heterocyclic group with two adjacent nitrogens, one of the nitrogen atoms of this group orients similarly to dCA in our docking analysis, and the adjacent nitrogen atom precludes the formation of a hydrogen bond with the backbone residues from the N terminus of Tat. Download FIG S6, PDF file, 0.1 MB. Copyright © 2019 Mediouni et al. 2019 Mediouni et al. This content is distributed under the terms of the Creative Commons Attribution 4.0 International license.

### dCA specifically inhibits the functions of the basic domain of Tat but not of Rev or Hexim-1.

**FIG 3:**
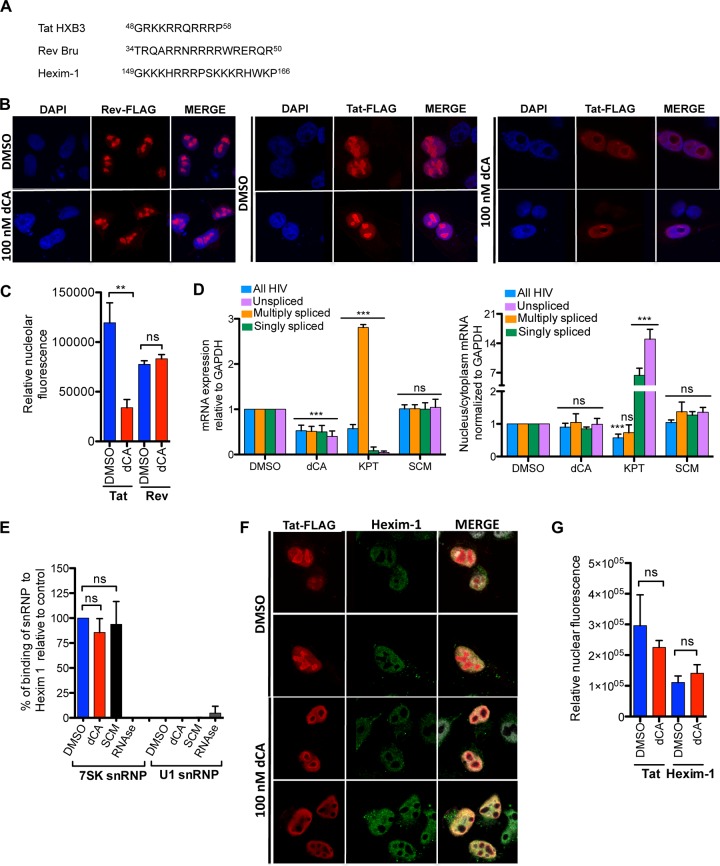
dCA does not interfere with Rev and Hexim-1 functions. (A) Sequence of the basic domains of HIV Tat HXB3, HIV Rev Bru and intracellular Hexim-1. (B) dCA does not exclude Rev from the nucleolus. Shown is confocal microscopy analysis of the subcellular localization of transfected FLAG-Tat or FLAG-Rev in the presence of DMSO or dCA in HeLa-CD4-LTR-Luc cells. Proteins were recognized with anti-FLAG and Alexa Fluor 568-tagged anti-IgG. Magnification 60×. Data are representative of *n* = 2 independent experiments. (C) Quantification of the nucleolar fluorescence of panel B using ImageJ. Data are the mean ± SEM from 3 to 4 different cells. (D) dCA does not perturb the HIV RNA cytoplasmic export function of HIV Rev. HeLa-CD4-LTR-LacZ cells were infected with strain NL4-3 for 24 hours. The next day, compounds (dCA, 10 nM; KPT, 400 nM; and SCM, 10 nM) were added for 24 hours. Total, nuclear, and cytoplasmic mRNAs were extracted, and viral messages were determined by qRT-PCR. GAPDH was used for normalization. Data are the mean ± SD from *n* = 5 independent experiments. (E) dCA does not interfere with the binding of Hexim-1 with 7SK snRNP. HeLa-CD4-LTR-LacZ cells were incubated with the indicated compounds. Forty-eight hours later, ChIP RNA with anti-Hexim antibody was performed. U1 snRNP, an unrelated RNA, and RNase treatment were used as controls. Data are the mean ± SD from *n* = 3 independent experiments. (F) dCA does not exclude intracellular Hexim-1 from the nucleus. Shown is confocal microscopy analysis of the subcellular localization of Hexim-1 in the presence of DMSO or dCA in HeLa-CD4-LTR-Luc cells. Magnification 60×. (G) Quantification of nuclear fluorescence of panel F using ImageJ. Data are the mean ± SEM from 3 to 6 different cells. Statistical significance was determined using one-way ANOVA with *post hoc* Tukey’s test. ns, not significant; ***, *P* < 0.0001; **, *P* < 0.001.

If dCA were to bind the basic domain of Tat in its disordered form, then one would predict it might also bind to other basic peptide sequences and block their functions. The two well-known proteins that share a similar basic domain with Tat are HIV-1 Rev protein and the cellular protein Hexim-1 (Fig. 3A). As such, we investigated whether dCA could inhibit these similar basic domain functions. The basic domain of Rev is responsible for its nuclear localization (31); therefore, we assessed whether dCA could impact the subcellular localization of Rev (Fig. 3B) as previously shown for Tat (10). We transfected HeLa-CD4-LTR-Luc cells with a plasmid expressing FLAG-tagged Rev and assessed Rev localization in the presence of 100 nM dCA. As expected for Tat used as the positive control, dCA caused a significant redistribution of Tat to the periphery of the nucleolus (Fig. 3B and C), forming a distinctive ring-like structure; however, the localization of Rev was left unchanged (Fig. 3B and C). Next, we investigated whether dCA was affecting the viral mRNA export function of Rev (Fig. 3D; Fig. S7A in the supplemental material). As such, we infected HeLa-CD4-LTR-LacZ cells with the NL4-3 strain and treated the cells with dCA or control compounds (KPT or SCM). KPT is an inhibitor of CRM1-mediated nuclear export. Twenty-four hours later, cells were collected, and total, nuclear, and cytoplasmic mRNAs were extracted, reverse transcribed, and quantified by quantitative real-time PCR (qRT-PCR). In whole-cell lysates (Fig. 3D, left), dCA treatment reduced all viral transcripts produced, as expected from a specific HIV-1 transcriptional inhibitor. In the presence of KPT, unspliced and singly spliced viral mRNAs were reduced, while the multiply spliced 2-kb viral mRNA species were increased (as previously shown in HeLa cells [32]). SCM, an unrelated compound, had no effect on HIV mRNAs. When analyzing the ratio of nuclear versus cytoplasmic viral mRNAs (Fig. 3D, right), KPT treatment resulted in a drastic accumulation of singly spliced and unspliced viral mRNAs in the nucleus compared to the dimethyl sulfoxide (DMSO) control, while SCM had no activity. dCA treatment did not mediate nuclear retention of singly or unspliced viral mRNAs as observed for KPT. We confirmed these results using the human T4 lymphoblastoid cell line CEM-SS (Fig. S7A). Together our results demonstrate that dCA does not impact Rev’s basic domain functions.

10.1128/mBio.02662-18.8 FIG S7 (A) dCA does not perturb the export function of HIV Rev in CEM-SS cells. Cells were infected with the NL4-3 strain for 24 hours. The next day, compounds (dCA, 30 nM; KPT, 600 nM; and SCM, 30 nM) were added for 24 hours. Total, nuclear, and cytoplasmic mRNAs were extracted, and viral messages were measured by qRT-PCR. GAPDH was used for normalization. Data are the mean ± SEM from *n* = 3 independent experiments. Statistical significance was determined using one-way ANOVA with *post hoc* Tukey’s test, comparing the DMSO condition to the other conditions. ***, *P* < 0.0001; **, *P* < 0.001; *, *P* < 0.01. (B) Measure of the impact of CDK8 knockdown on HIV expression. The anti-HIV activity of dCA, in acute infection of HeLa CD4 cells, is independent of CDK8. HeLa CD4 cells were transduced with VLPs expressing shRNAs against CDK8, CDK19, or both and selected with puromycin to stabilize shRNAs expression. Cells were then infected with NL4-3 strain for 24 hours, in the presence of DMSO or dCA (200 nM). After 24 hours, cells were washed, and fresh medium with compounds was added. Seventy hours later, viability was measured. Shown is the mean ± SEM from *n* = 3 independent experiments. Download FIG S7, PDF file, 0.1 MB. Copyright © 2019 Mediouni et al. 2019 Mediouni et al. This content is distributed under the terms of the Creative Commons Attribution 4.0 International license.

Most cellular P-TEFb is found in an inactive form in complex with the 7SK small nuclear ribonucleoprotein (snRNP) (33, 34), a tridimensional 4-stem-loop structure that serves as an RNA scaffold, among other proteins, for the homodimer of the CDK9-inhibitory protein Hexim-1/2. Hexim-1 directly binds to cyclin T1 and is responsible for the inactivation of the kinase activity of P-TEFb (35, 36). One proposed mechanism for P-TEFb release from 7SK snRNP is the direct competition between Tat and Hexim-1 for binding cyclin T1 and/or 7SK snRNP (reviewed in references 37 to 39). Given the high homology between Tat’s basic domain and Hexim-1, this basic region could also interfere with Hexim-1 association with 7SK snRNP by binding to a TAR-like motif within stem-loop 1 of 7SK snRNP (34, 40, 41). As such, we investigated whether dCA could interfere with the association of the basic domain of Hexim-1 with 7SK snRNP. Intracellular Hexim-1 was immunoprecipitated, and 7SK snRNP bound to Hexim-1 was extracted, reverse transcribed, and quantified by qRT-PCR as previously described (42) (Fig. 3E). HeLa-CD4-LTR-LacZ cells were treated with DMSO, dCA, or SCM (used as a negative control). The interaction between Hexim-1 and 7SK snRNP was similarly detected in the presence of DMSO, dCA, or SCM, but not when samples were subjected to RNase treatment, which was used as a positive control. The specificity of the binding of 7SK snRNP to Hexim-1 was also confirmed by the absence of U1 snRNP in the samples, used as a control. In parallel, we assessed the activity of dCA on Hexim-1 subcellular localization, but as expected, dCA did not have any effect on Hexim-1 nuclear localization (Fig. 3F and G). Altogether, our results suggest that dCA does not interfere with the activity of Rev or the binding of Hexim-1 to 7SK snRNP, reinforcing dCA’s specificity for the basic domain of Tat. This further supports our model that dCA is not affecting any stretch of poly-arginine residues, but rather interacts with the Tat basic patch in the context of the Tat structure.

### dCA activity on HIV-1 transcription is independent of CDK8.

The Mediator complexes are considered to be an integral part of the preinitiation complex by interacting with proteins, including RNAPII, general transcription factors, and transcription elongation factors, thus, serving as a signaling hub capable of integrating multiple inputs for precise control of RNAPII (43). In mammals, the Mediator is a large complex composed of 25 to 30 subunits organized into four distinct modules referred as the head, the middle, the tail, and the kinase module. The kinase module, consisting of CDK8, or its paralog CDK19, cyclin C, and two additional subunits, Med12 and Med13, carries the only known enzymatic activity in the complex and associates with the rest of the complex in a facultative fashion (44). Although the kinase module was initially characterized as a repressor, recent work has shown that it is also required for gene activation across multiple transcriptional programs (45). Several phosphorylation targets have been identified, including the RNAPII C-terminal domain (CTD) (45).

**FIG 4:**
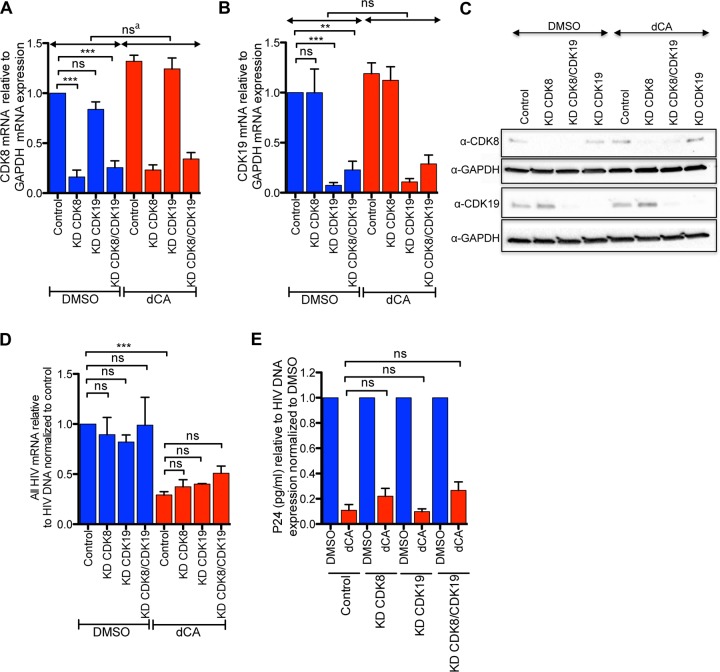
CDK8 does not regulate HIV-1 infection, and the activity of dCA is independent of CDK8. HeLa CD4 cells were transduced with VLPs expressing shRNAs against CDK8, CDK19, or both and puromycin selected to stabilize knockdown (KD). These cells were then infected with the NL4-3 strain for 24 h, in the presence of DMSO or dCA (200 nM). After 24 hours, cells were washed and fresh medium with compounds was added. Seventy hours later, mRNA, DNA, and p24 capsid in supernatant were collected and measured. (A and B) CDK8 mRNA and CDK19 mRNA expression was assessed by qRT-PCR and normalized to GAPDH mRNA. Shown is the mean ± SEM from *n* = 3 independent experiments. (C) Protein expression of CDK8 and CDK19 was analyzed 72 hours postinfection by Western blotting. GAPDH was used as an internal control. The image shown is representative of *n* = 3 independent experiments. (D) Viral mRNA production measured by qRT-PCR using primers against the Nef region was measured as a function of total integrated proviral DNA. Data represent the mean ± SEM from *n* = 3 independent experiments. (E) p24 capsid production in the supernatant relative to total HIV DNA and normalized to the DMSO of each condition is represented. Data represent the mean ± SEM from *n* = 3 independent experiments. Statistical significance was determined using one-way ANOVA with *post hoc* Tukey’s test. ns, not significant; ns^a^, DMSO KD CDK19 versus dCA KD CDK19. *, *P* < 0.01; ***, *P* < 0.0001; **, *P* < 0.001.

The only other known ligand for dCA is CDK8. Although the characteristics necessary for the antiretroviral activity of dCA do not overlap features needed for CDK8 inhibitory activity, it was important to verify whether the activity of dCA on HIV transcription involved CDK8 activity. As such, virus-like particles (VLPs) expressing short hairpin RNAs (shRNAs) against CDK8, CDK19, CDK8/CDK19, or the shRNA control were transduced into HeLa CD4 cells. Stabilization of the knockdown was performed by puromycin selection of the populations over a 2-week period. Both RT-qPCR and Western blotting confirmed successful knockdown of CDK8/CDK19 proteins (Fig. 4A and B). These cells were then infected with NL4-3 and treated or not with dCA, and production of viral RNA and p24 in the supernatant were assessed 72 hours later. While the viability of the cells remained unchanged (Fig. S7B), reduced expression of CDK8 and/or CDK19 did not significantly affect viral mRNA expression (Fig. 4D) and p24 capsid production, nor interfered with the antiviral activity of dCA (Fig. 4D and E). Our results are supported by prior report by Riuz and colleagues (46), which did not observe differences in viral replication upon knockdown of CDK8-associated Med12 and Med13 proteins. Moreover, work performed by the Karn group (47) demonstrated the mediator/CDK8 module is associated with the promoter during latency. Activation of NF-κB by tumor necrosis factor alpha (TNF-α) causes CDK8 to dissociate from the mediator, resulting in recruitment of TFIIH followed by RNAPII CTD phosphorylation and elongation. The mediator/CDK8 is lost during transcriptional activation, and thus, seemingly a marker of latency. As such, if dCA was to inhibit CDK8 in the context of HIV, one would expect activation of transcription rather than inhibition. Altogether, dCA does not mediate its anti-HIV activity through CDK8 inhibition, but rather through blocking Tat activity.

### dCA binding stabilizes Tat structure.

Our results suggest that dCA strengthens the intramolecular interactions between the basic and the N-terminal domains of Tat, promoting a more stable structure. As such, we assessed whether dCA increased Tat stability. First, we verified whether Tat when bound to dCA maintained proper folding by confirming that it was recognized by the conformational monoclonal antibody (MAb) 7G12 (48). Using competitive enzyme-linked immunosorbent assays (ELISAs), we incubated different ratios of dCA with Tat protein on an ELISA plate. In parallel, the plate was coated with Tat first, and dCA was added the following day. In both cases, the antibody at an equimolar ratio to Tat was added afterwards, followed by absorbance measurement. The MAb 7G12 was at limiting concentrations when dCA was added at higher molar ratios than Tat. Similar binding of MAb 7G12 to Tat protein was observed in the absence or presence of increasing concentrations of dCA (Fig. S8A). In pulldown assays followed by high-performance liquid chromatography (HPLC), Tat was first incubated with MAb 7G12, and then dCA was added to the complex before glycine HCl buffer elution and HPLC analysis with a C_8_ reverse-phase column (Fig. S8B). dCA’s peak was observed only when MAb 7G12-labeled Tat was present in the sample. We did not observe a peak of Tat because of the 10- to 20-µg detection threshold of the C_8_ HPLC column. The MAb 7G12’s peak was also not visible due to the cutoff of the C_8_ column or to the precipitation of the antibody in the salt-free elution buffer. Collectively, these results suggest that (i) Tat maintains proper folding since it binds a conformational epitope-recognizing antibody, and (ii) dCA bound to Tat does not occlude surface-exposed residues of the three-dimensional Tat, believed to be targeted by MAb 7G12 (^1^MEPV^4^, ^44^GISYGRKK^51^, and ^99^PED^101^) (27). In addition, since MAb 7G12 recognizes and neutralizes Tat from several variants, presenting small local structure variations (2), the changes of Tat structure promoted by dCA do not seem significantly important to prevent Tat recognition by MAb 7G12.

10.1128/mBio.02662-18.9 FIG S8 dCA recognizes folded Tat in solution. (A) dCA does not interfere with the interaction of Tat with MAb 7G12 by ELISA. MAb 7G12 is an antibody recognizing a tertiary structure of Tat. dCA was either preincubated with Tat and then the mixture was coated on a 96-well plate, or dCA was added after the blocking step (“dCA in solution”). Data are represented as the ratio of the signal of the MAb 7G12 bound to Tat under each condition relative to control signal without dCA. Data are the mean ± SD from *n* = 2 independent experiments. (B) dCA recognizes folded Tat after being pulled down with MAb 7G12. The MAb 7G12 was used at an equimolar concentration with Tat. dCA was then added to the mixture, and HPLC was used to reveal the interaction of Tat with dCA. Data are representative of *n* = 3 independent experiments. Download FIG S8, PDF file, 0.3 MB. Copyright © 2019 Mediouni et al. 2019 Mediouni et al. This content is distributed under the terms of the Creative Commons Attribution 4.0 International license.

**FIG 5:**
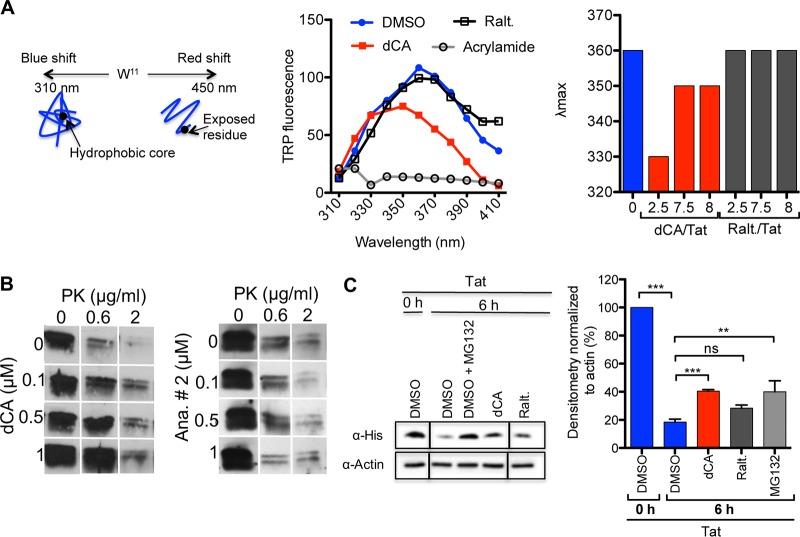
dCA stabilizes Tat structure. (A) Trp fluorescence assays. (Left) Schematic of the assay. (Center) DMSO, dCA (7.5× Tat concentration) or raltegravir (Ralt.; 7.5× Tat concentration) was incubated with Tat. The fluorescence of the Trp^11^ of Tat protein was measured at the indicated wavelengths. Acrylamide (250 mM) was used as a quenching control of Tat fluorescence. (Right) Peak absorbance (λ_max_) versus increasing fold ratios of compounds to Tat protein. Data are representative of *n* = 3 independent experiments. (B) DARTS assays. Tat was incubated with DMSO or dCA, at increasing concentrations, in the presence of increasing concentrations of proteinase K (PK). The protection was revealed by Western blotting. Analog 2 (Ana. # 2), an inactive analog of dCA (Table 2), was used as a negative control. Data are representative of *n* = 2 independent experiments. (C) Steady state of Tat in the presence of dCA *in vivo*. HeLa-CD4-LTR-Luc cells were incubated with recombinant Tat and then washed and incubated with the indicated compounds for 6 hours. The protection of Tat from degradation was revealed by assessing the amount of protein left by Western blotting. (Left) Western blot representative of *n* = 4 independent experiments. MG132 was used as a positive control of Tat protection from degradation, and raltegravir was used as a negative control. (Right) Densitometry of the Tat band normalized to the actin band. Results represent the mean ± SEM from *n* = 4 independent experiments. Statistical significance was determined using one-way ANOVA with *post hoc* Tukey’s test. ns, not significant; ***, *P* < 0.0001; **, *P* < 0.001.

Next, we investigated whether dCA mediated conformational changes in Tat. NMR studies have reported the fluorescence of Tat’s unique Trp^11^ to be highly sensitive to conformational modifications (2, 15). As such, we assessed Tat fluorescence between wavelengths of 310 nm and 410 nm (Fig. 5A, left). As reported, a λ_max_ was observed at 360 nm, and this fluorescence was quenched with acrylamide, used as control. The addition of dCA to Tat (dCA/Tat ratio of 7.5/1) resulted in the following changes: (i) decreased intensity of the Trp fluorescence, (ii) a reduction in λ_max_ (from 360 to 350 nm), and (iii) an increased *I*_330/350_ ratio with dCA (*I*_330/350_ = 0.893) compared to Tat alone (*I*_330/350_ = 0.73). Altogether these changes suggest that the presence of dCA amplified the optimal hydrophobic environment for Trp^11^. Raltegravir (raltegravir/Tat ratio of 7.5/1), used as a negative control, had no effect on the intrinsic fluorescence of Tat. To confirm these results, λ_max_ was measured at additional ratios of dCA to Tat protein and similar results were observed (Fig. 5A, right). In sum, these results suggest that dCA promotes burying of Trp^11^ in the optimal hydrophobic environment in the core of Tat, resulting in the stabilization of the protein.

To confirm that dCA stabilizes Tat structure, we performed drug affinity responsive target stability (DARTS) assays to measure the protection against proteolysis conferred on the target protein by interaction with a small molecule. For this purpose, we incubated Tat with several concentrations of dCA and different concentrations of proteinase K (PK) for 20 min at room temperature. PK digests proteins preferably after hydrophobic, aliphatic, and aromatic residues. The protection was determined by Western blotting with an anti-His antibody (Fig. 5B). Tat alone was proportionally degraded with increasing concentrations of PK. When the concentration of dCA was increased, Tat degradation by PK was significantly reduced. The inactive analog, analog 2 (Table 2), used as a negative control, did not protect Tat from degradation by PK, further supporting the molecular modeling results. Both the fluorescence assays and the DARTS assays support the notion that dCA stabilizes Tat protein.

Next, we determined the half-life of recombinant Tat in HeLa-CD4-LTR-Luc cells in the presence of dCA (Fig. 5C). We incubated recombinant Tat with cells for 4 hours, and after several washes to remove extracellular Tat protein, we incubated cells with DMSO, dCA, or raltegravir for an additional 6 hours. Western blotting with anti-His antibody was performed to reveal intracellular Tat. Tat protein levels after 6 hours were reduced by more than 80%, which is in agreement with previous reports (49). dCA significantly protected Tat from degradation, increasing the percentage of remaining Tat from 18% in DMSO to 40% with dCA. Raltegravir, used as a negative control, did not significantly alter Tat intracellular concentration (28%). The proteasome inhibitor MG132, used as a positive control, confirmed the previously reported involvement of the proteasome in Tat degradation (49, 50). dCA protected Tat from degradation to the same extent as MG132 (40%). Altogether, these results suggest that dCA may increase the steady-state level of Tat in cells by stabilizing Tat structure.

### dCA blocks Tat-TAR interaction.

**FIG 6:**
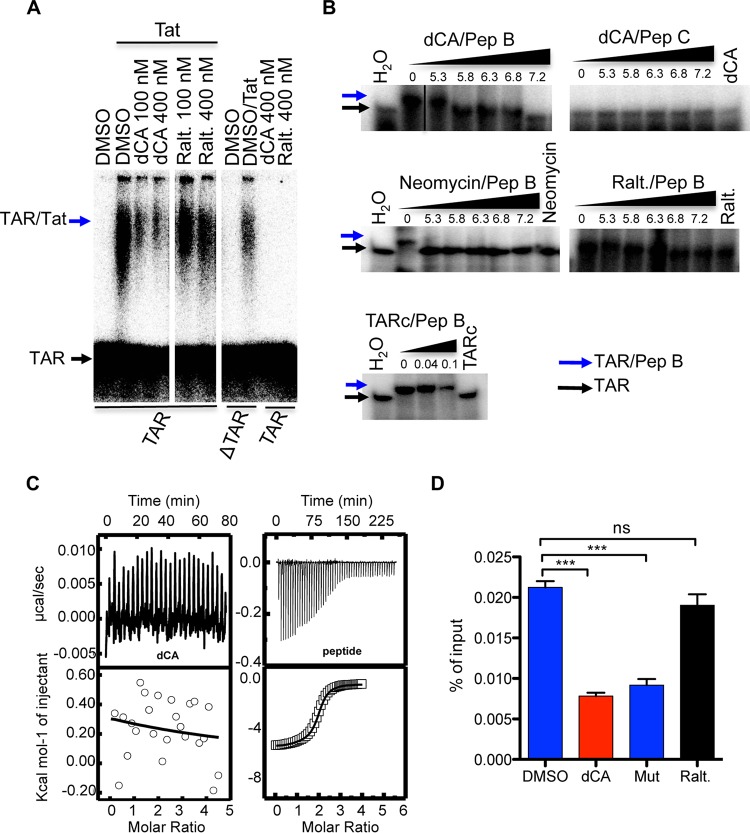
dCA binding to Tat disrupts Tat-TAR interaction. (A) EMSA of full-length Tat with TAR in the presence of dCA. Raltegravir (Ralt.) and deleted bulge TAR (ΔTAR) were used as negative controls. Data are representative of *n* = 3 independent experiments. (B) EMSA of Tat’s basic domain with TAR in the presence of increasing concentrations of dCA to Tat’s basic peptide. Pep B is a peptide encompassing the basic domain of HIV Tat (^45^ISYGRKKRRQRRRAP^59^). Pep C (^69^LSKQPASQPRGDPTG^83^) and unlabeled or cold TAR (TARc) were used as negative controls. Neomycin, which binds to Tat and TAR, was used as a positive control. Ratios of compounds (or TARc) concentrations to peptide are shown. Data are representative of *n* = 3 independent experiments. (C) dCA does not bind to TAR in ITC assays. Shown are ITC results from binding of dCA (left) or Tat peptide (right) to TAR at 25°C. In each panel, the top plot shows the baseline corrected experimental data. For the lower plots, results were converted to molar heats and plotted against the molar ratio of dCA or peptide to RNA. Data are representative of *n* = 2 independent experiments. (D) Tat-TAR interaction blocked by dCA in HeLa-CD4-LTR-Luc cells *in vivo*. Cells transfected with Tat or Tat Mut (arginine residues mutated to alanine in Tat’s basic domain) were incubated with DMSO or the indicated compounds (25 nM). Forty-eight hours later, cells were either lysed to determine the luciferase activity or cross-linked for ChIP assays. Luciferase activity was determined relative to protein concentration (Bradford assay) (Fig. S9A). Western blotting was performed to verify the amount of Tat under each condition (Fig. S9B). ChIP of Tat was followed by qRT-PCR of TAR DNA. The results are presented as percentage of input. The mean ± SEM is represented. Data are representative of *n* = 3 independent experiments. Statistical significance was determined using one-way ANOVA with *post hoc* Tukey’s test with 3 technical replicates. ns, not significant; ***, *P* < 0.0001.

Tat amplifies viral mRNA expression by binding through its basic domain to TAR, the 5′-terminal region stem-bulge-loop structure of HIV mRNAs, and recruits several cofactors to the viral promoter to promote transcriptional elongation. We investigated whether dCA could compete with TAR for Tat binding. For this purpose, we performed EMSA with radiolabeled TAR and recombinant Tat in the presence or absence of dCA (Fig. 6A). Raltegravir and a version of TAR with the Tat binding bulge deleted (ΔTAR) were used as negative controls. dCA inhibited the interaction of Tat with TAR in a dose-responsive manner, up to the same degree as the background determined by the nonspecific interaction of Tat with ΔTAR. This nonspecific interaction was previously reported, revealing two binding sites of Tat on TAR: a specific and high-affinity binding site and a nonspecific and low-affinity binding site (51). Raltegravir, used as a negative control, had no inhibitory activity.

10.1128/mBio.02662-18.10 FIG S9 Tat-TAR interaction blocked by dCA in HeLa-CD4-LTR-Luc cells. (A) Transactivation assays. HeLa-CD4-LTR-Luc cells, transfected with Tat or Tat Mut, were incubated with the indicated compounds (dCA and raltegravir [“Ralt.”], 25 nM) or DMSO. Forty-eight hours later, cells were lysed and cross-linked, and luciferase activity relative to the Bradford assay or ChIP TAR RNA (Fig. 6D) was determined. Data are the mean ± SD from *n* = 3 independent experiments. (B) Western blot of the amount of FLAG-Tat bound to FLAG-tagged beads after elution of the samples for ChIP TAR RNA, as control input. The input of FLAG-Tat and GAPDH as a loading control. Data are representative of *n* = 3 independent experiments. Statistical significance was determined using one-way ANOVA with *post hoc* Tukey’s test, comparing DMSO to the other conditions. ***, *P* < 0.0001. Download FIG S9, PDF file, 0.2 MB. Copyright © 2019 Mediouni et al. 2019 Mediouni et al. This content is distributed under the terms of the Creative Commons Attribution 4.0 International license.

We also performed the same experiment with a peptide encompassing the basic domain of Tat (Pep B; residues 45 to 59) and dCA. In this experiment, as negative controls we used a peptide of the C terminus of Tat (Pep C; residues 69 to 83), neomycin (a nonspecific inhibitor of Tat and TAR) (52), and unlabeled cold TAR (TARc) (Fig. 6B). dCA inhibited the interaction of Pep B with TAR in a dose-dependent manner, while Pep C did not interact with TAR and raltegravir did not compete with the Pep B-TAR interaction. As expected, both neomycin and TARc inhibited the interaction of Pep B with TAR. Using ITC, we also confirmed that dCA does not bind to TAR (Fig. 6C, left) as opposed to the basic peptide of Tat (Fig. 6C, right).

To confirm these results, we carried out a chromatin immunoprecipitation (ChIP) assay of TAR RNA on HeLa-CD4-LTR-Luc cells, upon transfection with FLAG-Tat or FLAG-Tat mutant (Mut) proteins in the presence or absence of dCA. We confirmed that dCA inhibited Tat transactivation activity by measuring the luciferase activity, and FLAG-Tat and FLAG-Tat Mut were well expressed under the different conditions (Fig. S9). Chromatin was immunoprecipitated with an anti-FLAG antibody, and qRT-PCR was performed to detect the presence of chromatin-coding TAR (Fig. 6D). A Tat-TAR interaction was detected in the presence of DMSO or raltegravir used as a negative control. However, dCA reduced this Tat-TAR interaction to similar levels as FLAG-Tat Mut, which aspecifically interacts with TAR. Altogether, our *in vitro* and *in vivo* results demonstrate that dCA blocks Tat transactivation by inhibiting Tat binding to TAR, completing our previous reports demonstrating dCA activity only on viruses with a competent Tat/TAR feedback loop (11).

## DISCUSSION

In an effort to determine the residues of Tat involved in the interaction with dCA and the molecular determinants of dCA activity, we synthetized 11 analogs of dCA and determined their activity in cell-based assays (Table 2). The inability to perform direct NMR studies of Tat with dCA was limited by the acidic formulation of dCA and the sensitivity of Tat structure to pH. As such, molecular modeling of dCA and analogs was possible by using available NMR structures of HIV Tat proteins present in PDB (1TBC, 1TIV, 1JFW, and 1K5K) (Tables 1 and 2 and Fig. 2; Fig. S3 and S4). Historically, it has been difficult to crystalize Tat due to its overall flexibility or lack of secondary structure, possibly explaining why only two of the NMR structures present in the PDB (1K5K and 1JFW, HIV-1 clades D and B, respectively) present fully exposed basic domains. The folding displayed by these two Tat structures was confirmed in studies of Tat entry in the endosomal compartment or Tat secretion (15, 17, 28, 53). The electrostatic interaction between Asp^2^ and two residues of the basic domain Arg^53^ and Lys^51^ for stabilization was also verified by mutagenesis and molecular dynamics simulations (14). Our molecular modeling combined with structure-function relationship studies suggests that the binding of dCA to Tat’s basic domain strengthens the intramolecular interactions between the basic and the N-terminal domains of Tat. In particular, the isoquinoline group of dCA may form hydrogen bonds with Arg^55^ and Pro^3^, and the hydroxyl groups of dCA may interact with Lys^51^ and Arg^52^ (Tables 1 and 2 and Fig. 2; Fig. S3 and S4). These residues correlate with Tat’s activity and structure (16). Tat entry in the endosomal compartment and Tat secretion require the exposure, under certain conditions (acidic pH or interaction with PI(4,5)P_2_), of the hidden Trp^11^ residue and the exposed basic domain of Tat protein. Both our Trp fluorescence and protection from proteolysis data support the concept that Tat has some intrinsic, if transient, structure that is stabilized by dCA (Fig. 5). dCA seemingly interacts with a specific Tat conformed form and not with a disordered form, since dCA does not bind to other proteins with similar basic patches (Fig. 3; Fig. S7A).

The analogs of dCA revealed key activity features such as the position of the nitrogen atom in the isoquinoline group of dCA, the hydroxyl groups of the estrone group, and the presence of the cycloheptene. The oxidation of C_16_ greatly improved the therapeutic index of dCA by increasing CC_50_, as observed with analog 9, all while abolishing dCA’s activity on its only other known ligand, CDK8. This result was expected since CDK8/CDK19 knockdown did not affect HIV-1 transcription (Fig. 4; Fig. S7B). Altogether, our work demonstrates that locking one conformer and blocking multiple binding interactions are a robust mechanism by which dCA achieves remarkable efficacy in driving long-term viral latency and limiting viral rebound upon treatment interruption (11, 12). Such efficacy results from the combined effects of dCA on (i) blocking interaction of Tat with TAR (Fig. 6; Fig. S9), (ii) blocking extracellular Tat uptake by cells (13), and (iii) interfering with nucleolar localization (Fig. 3B) (10, 13). The mode of action of dCA is somewhat reminiscent of the activity of arginine methyltransferase (PRMT6) and the SET domain bifurcated 1 (SETDB1) protein. By binding to Tat residues Arg^52^ and Arg^53^, PRMT6 changes Tat’s structure, decreasing the affinity of Tat for TAR, all while increasing the stability of Tat in the cytoplasm (54). As for SETDB1, it methylates Tat’s Lys^50^ and Lys^51^ *in vitro*, inhibiting viral transcription (55).

Tat is an attractive target for therapeutic intervention because it is expressed early during viral replication, it has no cellular homologs, and direct inhibition of Tat blocks the feedback loop that drives exponential viral production. dCA penetrates viral sanctuaries such as the brain, has a drug-like structure, is very soluble in water, displays good bioavailability in mice, and acts additively with other antiretrovirals (10, 12, 13). The understanding of the molecular determinants for the activity of dCA on Tat reported here, provided by a combination of classic biophysical and biochemical approaches, is invaluable for the clinical development of the cortistatin A pharmacophore, and in general, for the development of inhibitors against Tat.

## MATERIALS AND METHODS

### Bacterial expression and purification of Tat.

Expression and purification of Tat were performed as previously described (13). A detailed protocol is provided in Text S1 in the supplemental material.

10.1128/mBio.02662-18.1 TEXT S1 Supplemental methods. Download Text S1, DOCX file, 0.2 MB. Copyright © 2019 Mediouni et al. 2019 Mediouni et al. This content is distributed under the terms of the Creative Commons Attribution 4.0 International license.

### Transactivation assay.

The activity of Tat in HeLa-CD4-LTR-Luc and OM10.1 cells was verified by a transactivation assay as described in reference 13. In HeLa-CD4-LTR-LacZ cells, the assay was performed as described in reference 10. A detailed protocol is provided in Text S1.

### Immunofluorescence.

HeLa-CD4-LTR-Luc cells were grown on fibronectin-treated coverslips and transfected with PGK (phosphoglycerate kinase 1)-FLAG-Tat or PGK-FLAG-Rev. Following transfection, cells were treated with DMSO or dCA (100 nM), and staining/imaging was performed as previously reported (10, 13). Anti-Hexim antibody (Abcam) was used to detect intracellular Hexim-1. ImageJ was used to quantify the relative fluorescence of the nucleolus or nucleus.

### Knockdown of CDK8/CDK19 in HeLa CD4 cells and effect on HIV replication.

shRNA CDK8#2-fwd (GATCCCCCCTCTGGCATATAATCAAGTTTTCAAGAGAAACTTGATTATATGCCAGAGGTTTTTA) and shRNA CDK8#2-rev (AGCTTAAAAACCTCTGGCATATAATCAAGTTTCTCTTGAAAACTTGATTATATGCCAGAGGGGG) (56) or shRNA CDK19 fwd (GATCCCCGTAGCTAAGTCTACCTTAATTCAAGAGATTAAGGTAGACTTAGCTACTTTTTA) and shRNA CDK19 rev (AGCTTAAAAAGTAGCTAAGTCTACCTTAATCTCTTGAATTAAGGTAGACTTAGCTACGGG) (57, 58) were annealed and ligated in the digested pSUPER.Retro.Puro vector with the BglII and HindIII restriction enzymes. The constructs were then cotransfected with the pMD-G and pHit60 vectors in HEK293 T cells to obtain VLPs. HeLa-CD4 cells were transduced with these VLPs. The following day, puromycin (Gemini) at 2 μg/ml was added to select shRNA-expressing cells. Knockdown of CDK8/CDK19 mRNA or protein expression was determined by qRT-PCR and Western blotting with an anti-CDK8 antibody (ab115155; Abcam) or anti-CDK19 antibody (HPA007053; Sigma-Aldrich) at a 1:1,000 dilution. CDK8/CDK19 knockdown HeLa-CD4-LTR-LacZ cells were then infected with NL4-3 in the presence of dCA (200 nM) or DMSO. The next day, cells were washed and fresh medium containing dCA or DMSO was added. Seventy hours later, cellular pellets and cellular supernatants were kept for future analyses.

### RNA extraction and qRT-PCR analysis.

RNA extraction and qRT-PCR were performed as previously described (59). Primer sequences for GAPDH (glyceraldehyde-3-phosphate dehydrogenase) (13) and all viral mRNAs, unspliced, singly spliced, and multiply spliced as in (59). Primer sequences for CDK8 and CDK19 were from references 57 and 58.

### EMSA.

EMSA with full-length Tat, a protocol similar to that of Das et al. was performed (60). Details on the EMSA with Tat’s basic peptide are provided in Text S1.

### Trp fluorescence.

Recombinant Tat (2.5 μM) was incubated with different concentrations of dCA or raltegravir in a 96-well plate. The solution was excited at 280 nm, and the emission of Trp fluorescence was read from 310 to 410 nm. The background induced by the compounds alone or buffer of dilution was subtracted from the Trp signal. Acrylamide (250 mM) was used as a quencher of the fluorescence.

### DARTS assay.

dCA or an analog of dCA (analog 2) was incubated with 30 ng of recombinant Tat for 20 min at room temperature. Then PK (Ambion) was added at different concentrations. The remaining Tat was quantified by Western blotting using polyclonal anti-Tat antibodies (AIDS Reagent Program) at a 1:2,000 dilution.

### Mitochondrial metabolic activity assay.

The assay was performed with MTT [3-(4,5-dimethyl-2-thiazolyl)-2,5-diphenyl-2H-tetrazolium bromide] as described in reference 10.

### RNA immunoprecipitation of 7SK snRNP.

The immunoprecipitation of the 7SK snRNP by intracellular Hexim-1 was performed as previously described in reference 42, with some modifications detailed in Text S1.

### Molecular docking of Tat-dCA.

Docking experiments were performed using AutoDock Vina (61). The HIV-1 Tat protein was used as a template for docking experiments (PDB entries 1K5K, 1JFW, 1TIV, and 1TBC). All the ligands were semi-empirically optimized using MOPAC (Molecular Orbital Package), and all the atomic charges were calculated and optimized using the AM1 method in VEGA.

### Isothermal titration calorimetry.

A detailed protocol for ITC is provided in Text S1.

### ChiP assay.

Performed as previously described (62); a detailed protocol is provided in Text S1.

### Statistical analysis.

Statistical analyses were performed with GraphPad Prism, and a *P* value of ≤0.05 was considered significant for all comparisons. The two-tailed paired Student's *t* test or one-way analysis of variance (ANOVA) repeated-measures test was used when applicable. Tukey’s correction was used for *post hoc* analysis.

## References

[1] Ptak RG, Fu W, Sanders-Beer BE, Dickerson JE, Pinney JW, Robertson DL, Rozanov MN, Katz KS, Maglott DR, Pruitt KD, Dieffenbach CW 2008 Cataloguing the HIV type 1 human protein interaction network. AIDS Res Hum Retroviruses 24:1497–1502. doi:10.1089/aid.2008.0113. 19025396 PMC2655106

[2] Campbell GR, Loret EP 2009 What does the structure-function relationship of the HIV-1 Tat protein teach us about developing an AIDS vaccine? Retrovirology 6:50. doi:10.1186/1742-4690-6-50. 19467159 PMC2693501

[3] Mediouni S, Darque A, Baillat G, Ravaux I, Dhiver C, Tissot-Dupont H, Mokhtari M, Moreau H, Tamalet C, Brunet C, Paul P, Dignat-George F, Stein A, Brouqui P, Spector SA, Campbell GR, Loret EP 2012 Antiretroviral therapy does not block the secretion of the human immunodeficiency virus Tat protein. Infect Disord Drug Targets 12:81–86. 22280310 10.2174/187152612798994939

[4] Karn J 2011 The molecular biology of HIV latency: breaking and restoring the Tat-dependent transcriptional circuit. Curr Opin HIV AIDS 6:4–11. doi:10.1097/COH.0b013e328340ffbb. 21242887 PMC3032057

[5] Mediouni S, Marcondes MC, Miller C, McLaughlin JP, Valente ST 2015 The cross-talk of HIV-1 Tat and methamphetamine in HIV-associated neurocognitive disorders. Front Microbiol 6:1164. doi:10.3389/fmicb.2015.01164. 26557111 PMC4615951

[6] Bechara C, Sagan S 2013 Cell-penetrating peptides: 20 years later, where do we stand? FEBS Lett 587:1693–1702. doi:10.1016/j.febslet.2013.04.031. 23669356

[7] Aoki S, Watanabe Y, Sanagawa M, Setiawan A, Kotoku N, Kobayashi M 2006 Cortistatins A, B, C, and D, anti-angiogenic steroidal alkaloids, from the marine sponge Corticium simplex. J Am Chem Soc 128:3148–3149. doi:10.1021/ja057404h. 16522087

[8] Aoki S, Watanabe Y, Tanabe D, Arai M, Suna H, Miyamoto K, Tsujibo H, Tsujikawa K, Yamamoto H, Kobayashi M 2007 Structure-activity relationship and biological property of cortistatins, anti-angiogenic spongean steroidal alkaloids. Bioorg Med Chem 15:6758–6762. doi:10.1016/j.bmc.2007.08.017. 17765550

[9] Pelish HE, Liau BB, Nitulescu II, Tangpeerachaikul A, Poss ZC, Da Silva DH, Caruso BT, Arefolov A, Fadeyi O, Christie AL, Du K, Banka D, Schneider EV, Jestel A, Zou G, Si C, Ebmeier CC, Bronson RT, Krivtsov AV, Myers AG, Kohl NE, Kung AL, Armstrong SA, Lemieux ME, Taatjes DJ, Shair MD 2015 Mediator kinase inhibition further activates super-enhancer-associated genes in AML. Nature 526:273–276. doi:10.1038/nature14904. 26416749 PMC4641525

[10] Mousseau G, Clementz MA, Bakeman WN, Nagarsheth N, Cameron M, Shi J, Baran P, Fromentin R, Chomont N, Valente ST 2012 An analog of the natural steroidal alkaloid cortistatin A potently suppresses Tat-dependent HIV transcription. Cell Host Microbe 12:97–108. doi:10.1016/j.chom.2012.05.016. 22817991 PMC3403716

[11] Mousseau G, Kessing CF, Fromentin R, Trautmann L, Chomont N, Valente ST 2015 The Tat inhibitor didehydro-cortistatin A prevents HIV-1 reactivation from latency. mBio 6:e00465-15. doi:10.1128/mBio.00465-15. 26152583 PMC4495168

[12] Kessing CF, Nixon CC, Li C, Tsai P, Takata H, Mousseau G, Ho PT, Honeycutt JB, Fallahi M, Trautmann L, Garcia JV, Valente ST 2017 In vivo suppression of HIV rebound by didehydro-cortistatin A, a “block-and-lock” strategy for HIV-1 treatment. Cell Rep 21:600–611. doi:10.1016/j.celrep.2017.09.080. 29045830 PMC5653276

[13] Mediouni S, Jablonski J, Paris J, Clementz M, Thenin-Houssier S, McLaughlin J, Valente S 2015 Didehydro-cortistatin A inhibits HIV-1 Tat mediated neuroinflammation and prevents potentiation of cocaine reward in Tat transgenic mice. Curr HIV Res 13:64–79. doi:10.2174/1570162X13666150121111548. 25613133 PMC4416414

[14] Pantano S, Carloni P 2005 Comparative analysis of HIV-1 Tat variants. Proteins 58:638–643. doi:10.1002/prot.20323. 15609368

[15] Yezid H, Konate K, Debaisieux S, Bonhoure A, Beaumelle B 2009 Mechanism for HIV-1 Tat insertion into the endosome membrane. J Biol Chem 284:22736–22746. doi:10.1074/jbc.M109.023705. 19549783 PMC2755682

[16] Pantano S, Tyagi M, Giacca M, Carloni P 2004 Molecular dynamics simulations on HIV-1 Tat. Eur Biophys J 33:344–351. doi:10.1007/s00249-003-0358-z. 14608449

[17] Debaisieux S, Rayne F, Yezid H, Beaumelle B 2012 The ins and outs of HIV-1 Tat. Traffic 13:355–363. doi:10.1111/j.1600-0854.2011.01286.x. 21951552

[18] Gu J, Babayeva ND, Suwa Y, Baranovskiy AG, Price DH, Tahirov TH 2014 Crystal structure of HIV-1 Tat complexed with human P-TEFb and AFF4. Cell Cycle 13:1788–1797. doi:10.4161/cc.28756. 24727379 PMC4111725

[19] Tahirov TH, Babayeva ND, Varzavand K, Cooper JJ, Sedore SC, Price DH 2010 Crystal structure of HIV-1 Tat complexed with human P-TEFb. Nature 465:747–751. doi:10.1038/nature09131. 20535204 PMC2885016

[20] Gregoire C, Peloponese JM Jr, Esquieu D, Opi S, Campbell G, Solomiac M 2001 Homonuclear (1)H-NMR assignment and structural characterization of human immunodeficiency virus type 1 Tat Mal protein. Biopolymers 62:324–335. doi:10.1002/bip.10000. 11857271

[21] Shojania S, Henry GD, Chen VC, Vo TN, Perreault H, O'Neil JD 2010 High yield expression and purification of HIV-1 Tat1-72 for structural studies. J Virol Methods 164:35–42. doi:10.1016/j.jviromet.2009.11.021. 19941902

[22] Siddappa NB, Venkatramanan M, Venkatesh P, Janki MV, Jayasuryan N, Desai A, Ravi V, Ranga U 2006 Transactivation and signaling functions of Tat are not correlated: biological and immunological characterization of HIV-1 subtype-C Tat protein. Retrovirology 3:53. doi:10.1186/1742-4690-3-53. 16916472 PMC1564039

[23] Butera ST, Perez VL, Wu BY, Nabel GJ, Folks TM 1991 Oscillation of the human immunodeficiency virus surface receptor is regulated by the state of viral activation in a CD4^+^ cell model of chronic infection. J Virol 65:4645–4653. 1678437 10.1128/jvi.65.9.4645-4653.1991 PMC248919

[24] Xiao H, Neuveut C, Tiffany HL, Benkirane M, Rich EA, Murphy PM, Jeang KT 2000 Selective CXCR4 antagonism by Tat: implications for in vivo expansion of coreceptor use by HIV-1. Proc Natl Acad Sci U S A 97:11466–11471. doi:10.1073/pnas.97.21.11466. 11027346 PMC17223

[25] Campbell GR, Senkaali D, Watkins J, Esquieu D, Opi S, Yirrell DL, Kaleebu P, Loret EP 2007 Tat mutations in an African cohort that do not prevent transactivation but change its immunogenic properties. Vaccine 25:8441–8447. doi:10.1016/j.vaccine.2007.09.070. 17997200

[26] Kuppuswamy M, Subramanian T, Srinivasan A, Chinnadurai G 1989 Multiple functional domains of Tat, the trans-activator of HIV-1, defined by mutational analysis. Nucleic Acids Res 17:3551–3561. 2542902 10.1093/nar/17.9.3551 PMC317795

[27] Mediouni S, Darque A, Ravaux I, Baillat G, Devaux C, Loret EP 2013 Identification of a highly conserved surface on Tat variants. J Biol Chem 288:19072–19080. doi:10.1074/jbc.M113.466011. 23678001 PMC3696680

[28] Rayne F, Debaisieux S, Bonhoure A, Beaumelle B 2010 HIV-1 Tat is unconventionally secreted through the plasma membrane. Cell Biol Int 34:409–413. doi:10.1042/CBI20090376. 19995346

[29] Watkins JD, Lancelot S, Campbell GR, Esquieu D, de Mareuil J, Opi S, Annappa S, Salles J-P, Loret EP 2006 Reservoir cells no longer detectable after a heterologous SHIV challenge with the synthetic HIV-1 Tat Oyi vaccine. Retrovirology 3:8. doi:10.1186/1742-4690-3-8. 16441880 PMC1434768

[30] Wang Z, Dai M, Park PK, Danishefsky SJ 2011 Synthetic studies toward (+)-cortistatin A. Tetrahedron 67:10249–10260. doi:10.1016/j.tet.2011.10.026. 22879684 PMC3413295

[31] Nam YS, Petrovic A, Jeong KS, Venkatesan S 2001 Exchange of the basic domain of human immunodeficiency virus type 1 Rev for a polyarginine stretch expands the RNA binding specificity, and a minimal arginine cluster is required for optimal RRE RNA binding affinity, nuclear accumulation, and trans-activation. J Virol 75:2957–2971. doi:10.1128/JVI.75.6.2957-2971.2001. 11222721 PMC115922

[32] Boons E, Vanstreels E, Jacquemyn M, Nogueira TC, Neggers JE, Vercruysse T, van den Oord J, Tamir S, Shacham S, Landesman Y, Snoeck R, Pannecouque C, Andrei G, Daelemans D 2015 Human exportin-1 is a target for combined therapy of HIV and AIDS related lymphoma. EBioMedicine 2:1102–1113. doi:10.1016/j.ebiom.2015.07.041. 26501108 PMC4588406

[33] Barboric M, Yik JHN, Czudnochowski N, Yang Z, Chen R, Contreras X, Geyer M, Matija Peterlin B, Zhou Q 2007 Tat competes with HEXIM1 to increase the active pool of P-TEFb for HIV-1 transcription. Nucleic Acids Res 35:2003–2012. doi:10.1093/nar/gkm063. 17341462 PMC1874611

[34] Sedore SC, Byers SA, Biglione S, Price JP, Maury WJ, Price DH 2007 Manipulation of P-TEFb control machinery by HIV: recruitment of P-TEFb from the large form by Tat and binding of HEXIM1 to TAR. Nucleic Acids Res 35:4347–4358. doi:10.1093/nar/gkm443. 17576689 PMC1935001

[35] Chen R, Yang Z, Zhou Q 2004 Phosphorylated positive transcription elongation factor b (P-TEFb) is tagged for inhibition through association with 7SK snRNA. J Biol Chem 279:4153–4160. doi:10.1074/jbc.M310044200. 14627702

[36] Li Q, Price JP, Byers SA, Cheng D, Peng J, Price DH 2005 Analysis of the large inactive P-TEFb complex indicates that it contains one 7SK molecule, a dimer of HEXIM1 or HEXIM2, and two P-TEFb molecules containing Cdk9 phosphorylated at threonine 186. J Biol Chem 280:28819–28826. doi:10.1074/jbc.M502712200. 15965233

[37] Quaresma AJC, Bugai A, Barboric M 2016 Cracking the control of RNA polymerase II elongation by 7SK snRNP and P-TEFb. Nucleic Acids Res 44:7527–7539. doi:10.1093/nar/gkw585. 27369380 PMC5027500

[38] Lu H, Li Z, Xue Y, Zhou Q 2013 Viral-host interactions that control HIV-1 transcriptional elongation. Chem Rev 113:8567–8582. doi:10.1021/cr400120z. 23795863 PMC4310557

[39] Mousseau G, Valente ST 2017 Role of host factors on the regulation of Tat-mediated HIV-1 transcription. Curr Pharm Des 23:4079–4090. doi:10.2174/1381612823666170622104355. 28641539 PMC5731639

[40] Muniz L, Egloff S, Ughy B, Jady BE, Kiss T 2010 Controlling cellular P-TEFb activity by the HIV-1 transcriptional transactivator Tat. PLoS Pathog 6:e1001152. doi:10.1371/journal.ppat.1001152. 20976203 PMC2954905

[41] Yik JH, Chen R, Pezda AC, Samford CS, Zhou Q 2004 A human immunodeficiency virus type 1 Tat-like arginine-rich RNA-binding domain is essential for HEXIM1 to inhibit RNA polymerase II transcription through 7SK snRNA-mediated inactivation of P-TEFb. Mol Cell Biol 24:5094–5105. doi:10.1128/MCB.24.12.5094-5105.2004. 15169877 PMC419863

[42] Bartholomeeusen K, Xiang Y, Fujinaga K, Peterlin BM 2012 Bromodomain and extra-terminal (BET) bromodomain inhibition activate transcription via transient release of positive transcription elongation factor b (P-TEFb) from 7SK small nuclear ribonucleoprotein. J Biol Chem 287:36609–36616. doi:10.1074/jbc.M112.410746. 22952229 PMC3476326

[43] Malik S, Roeder RG 2010 The metazoan Mediator co-activator complex as an integrative hub for transcriptional regulation. Nat Rev Genet 11:761–772. doi:10.1038/nrg2901. 20940737 PMC3217725

[44] Conaway RC, Conaway JW 2011 Function and regulation of the Mediator complex. Curr Opin Genet Dev 21:225–230. doi:10.1016/j.gde.2011.01.013. 21330129 PMC3086004

[45] Galbraith MD, Donner AJ, Espinosa JM 2010 CDK8: a positive regulator of transcription. Transcription 1:4–12. doi:10.4161/trns.1.1.12373. 21327159 PMC3035184

[46] Ruiz A, Pauls E, Badia R, Riveira-Muñoz E, Clotet B, Ballana E, Esté JA 2014 Characterization of the influence of mediator complex in HIV-1 transcription. J Biol Chem 289:27665–27676. doi:10.1074/jbc.M114.570341. 25100719 PMC4183804

[47] Kim YK, Bourgeois CF, Pearson R, Tyagi M, West MJ, Wong J, Wu S-Y, Chiang C-M, Karn J 2006 Recruitment of TFIIH to the HIV LTR is a rate-limiting step in the emergence of HIV from latency. EMBO J 25:3596–3604. doi:10.1038/sj.emboj.7601248. 16874302 PMC1538560

[48] Mediouni S, Watkins JD, Pierres M, Bole A, Loret EP, Baillat G 2012 A monoclonal antibody directed against a conformational epitope of the HIV-1 trans-activator (Tat) protein neutralizes cross-clade. J Biol Chem 287:11942–11950. doi:10.1074/jbc.M111.319863. 22362765 PMC3320942

[49] Zhao X, Qian L, Zhou D, Qi D, Liu C, Kong X 2016 Stability of HIV-1 subtype B and C Tat is associated with variation in the carboxyl-terminal region. Virol Sin 31:199–206. doi:10.1007/s12250-016-3681-0. 27007880 PMC8193441

[50] Sivakumaran H, van der Horst A, Fulcher AJ, Apolloni A, Lin M-H, Jans DA, Harrich D 2009 Arginine methylation increases the stability of human immunodeficiency virus type 1 Tat. J Virol 83:11694–11703. doi:10.1128/JVI.00499-09. 19726520 PMC2772670

[51] Morgner N, Barth HD, Brutschy B, Scheffer U, Breitung S, Gobel M 2008 Binding sites of the viral RNA element TAR and of TAR mutants for various peptide ligands, probed with LILBID: a new laser mass spectrometry. J Am Soc Mass Spectrom 19:1600–1611. doi:10.1016/j.jasms.2008.07.001. 18693035

[52] Wang S, Huber PW, Cui M, Czarnik AW, Mei HY 1998 Binding of neomycin to the TAR element of HIV-1 RNA induces dissociation of Tat protein by an allosteric mechanism. Biochemistry 37:5549–5557. doi:10.1021/bi972808a. 9548939

[53] Rayne F, Debaisieux S, Yezid H, Lin Y-L, Mettling C, Konate K, Chazal N, Arold ST, Pugnière M, Sanchez F, Bonhoure A, Briant L, Loret E, Roy C, Beaumelle B 2010 Phosphatidylinositol-(4,5)-bisphosphate enables efficient secretion of HIV-1 Tat by infected T-cells. EMBO J 29:1348–1362. doi:10.1038/emboj.2010.32. 20224549 PMC2868572

[54] Xie B, Invernizzi CF, Richard S, Wainberg MA 2007 Arginine methylation of the human immunodeficiency virus type 1 Tat protein by PRMT6 negatively affects Tat interactions with both cyclin T1 and the Tat transactivation region. J Virol 81:4226–4234. doi:10.1128/JVI.01888-06. 17267505 PMC1866113

[55] Van Duyne R, Easley R, Wu W, Berro R, Pedati C, Klase Z, Kehn-Hall K, Flynn EK, Symer DE, Kashanchi F 2008 Lysine methylation of HIV-1 Tat regulates transcriptional activity of the viral LTR. Retrovirology 5:40. doi:10.1186/1742-4690-5-40. 18498648 PMC2412914

[56] Porter DC, Farmaki E, Altilia S, Schools GP, West DK, Chen M, Chang B-D, Puzyrev AT, Lim C-U, Rokow-Kittell R, Friedhoff LT, Papavassiliou AG, Kalurupalle S, Hurteau G, Shi J, Baran PS, Gyorffy B, Wentland MP, Broude EV, Kiaris H, Roninson IB 2012 Cyclin-dependent kinase 8 mediates chemotherapy-induced tumor-promoting paracrine activities. Proc Natl Acad Sci U S A 109:13799–13804. doi:10.1073/pnas.1206906109. 22869755 PMC3427077

[57] Galbraith MD, Allen MA, Bensard CL, Wang X, Schwinn MK, Qin B, Long HW, Daniels DL, Hahn WC, Dowell RD, Espinosa JM 2013 HIF1A employs CDK8-mediator to stimulate RNAPII elongation in response to hypoxia. Cell 153:1327–1339. doi:10.1016/j.cell.2013.04.048. 23746844 PMC3681429

[58] Donner AJ, Ebmeier CC, Taatjes DJ, Espinosa JM 2010 CDK8 is a positive regulator of transcriptional elongation within the serum response network. Nat Struct Mol Biol 17:194–201. doi:10.1038/nsmb.1752. 20098423 PMC2920286

[59] Jablonski JA, Caputi M 2009 Role of cellular RNA processing factors in human immunodeficiency virus type 1 mRNA metabolism, replication, and infectivity. J Virol 83:981–992. doi:10.1128/JVI.01801-08. 19004959 PMC2612387

[60] Das AT, Klaver B, Harwig A, Vink M, Ooms M, Centlivre M, Berkhout B 2007 Construction of a doxycycline-dependent simian immunodeficiency virus reveals a nontranscriptional function of Tat in viral replication. J Virol 81:11159–11169. doi:10.1128/JVI.01354-07. 17670816 PMC2045552

[61] Trott O, Olson AJ 2010 AutoDock Vina: improving the speed and accuracy of docking with a new scoring function, efficient optimization, and multithreading. J Comput Chem 31:455–461. doi:10.1002/jcc.21334. 19499576 PMC3041641

[62] Kim HY, Choi BS, Kim SS, Roh TY, Park J, Yoon CH 2014 NUCKS1, a novel Tat coactivator, plays a crucial role in HIV-1 replication by increasing Tat-mediated viral transcription on the HIV-1 LTR promoter. Retrovirology 11:67. doi:10.1186/PREACCEPT-1444760432121123. 25116364 PMC4181878

