# Supplementary material for: Didehydro-Cortistatin A Inhibits HIV-1 by Specifically Binding to the Unstructured Basic Region of Tat
Source: mBio. 2019 Feb 5;10(1):e02662-18. doi: 10.1128/mBio.02662-18 (PMC6368365; doi:10.1128/mBio.02662-18)
Supplement: FIG S1 [file mBio.02662-18-sf001.pdf]

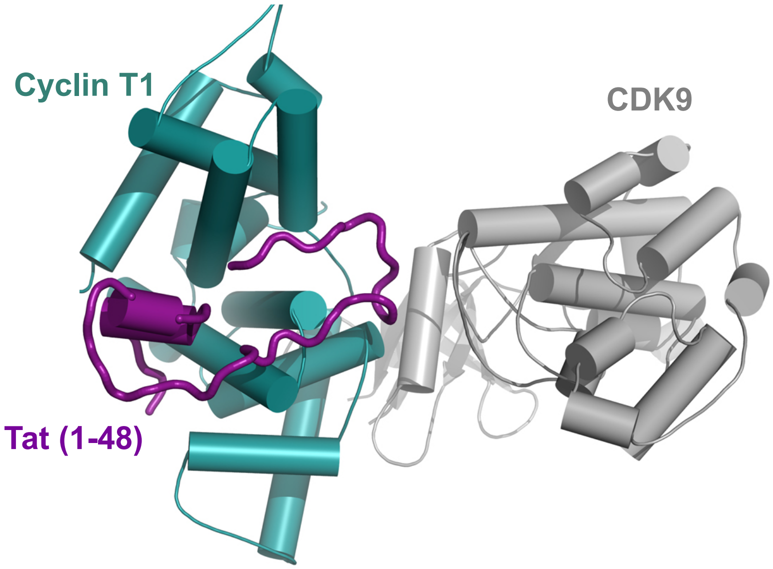

Figure S1. Crystal structure of HIV-1 Tat complexed with ATP-bound human P-TEFb. Residues 1 to 48 of Tat protein are resolved in the structure with visible α-helices for the C-terminus region (residues 28-43). Despite unstructured, the N-terminus of Tat protein is stabilized by the P-TEFb protein in the complex. Cyclin T1 is colored in dark green, CDK9 in grey and Tat in purple. This figure was prepared with data from PDB entry 3MIA.
