# Supplementary material for: Didehydro-Cortistatin A Inhibits HIV-1 by Specifically Binding to the Unstructured Basic Region of Tat
Source: mBio. 2019 Feb 5;10(1):e02662-18. doi: 10.1128/mBio.02662-18 (PMC6368365; doi:10.1128/mBio.02662-18)
Supplement: FIG S2 [file mBio.02662-18-sf002.pdf]

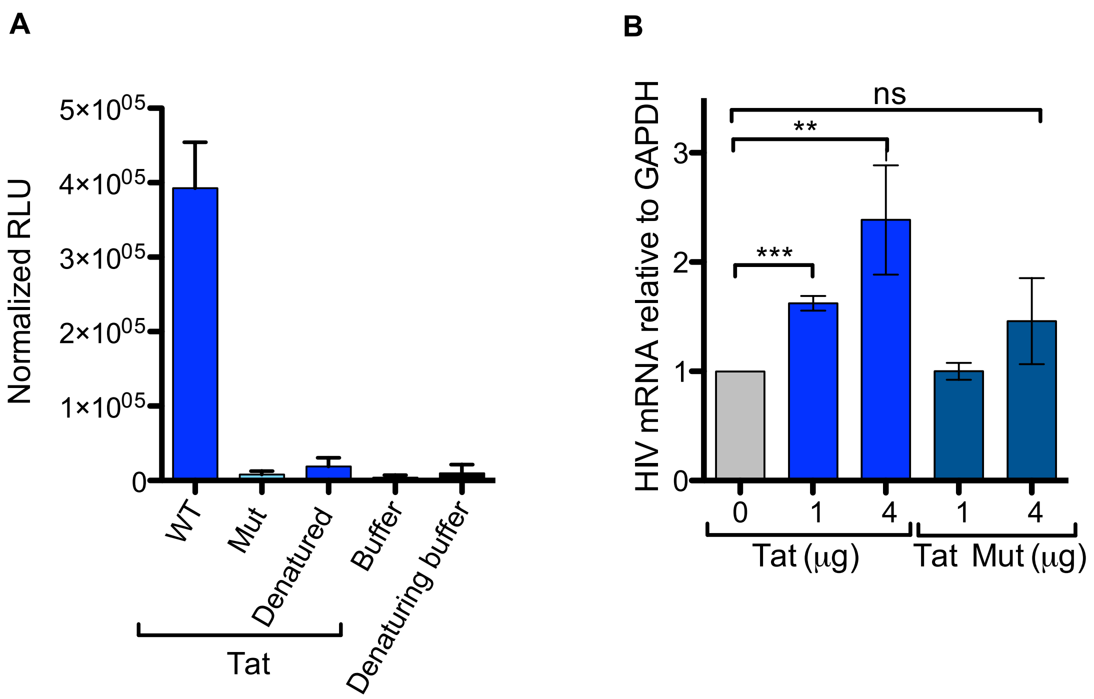

Figure S2. Tat transactivation activity. Transactivation activity in (**A**) HeLa-CD4-LTR-Luc cells and (**B**) OM10.1 cells. Relative light units (RLU) is luciferase per total protein. Data is the mean ± SD of n=3 independent experiments. Statistical significance was determined using one way Anova with post-hoc Turkey test, $p < 0.0001$: ***, $p < 0.001$: **.
