# Supplementary material for: Didehydro-Cortistatin A Inhibits HIV-1 by Specifically Binding to the Unstructured Basic Region of Tat
Source: mBio. 2019 Feb 5;10(1):e02662-18. doi: 10.1128/mBio.02662-18 (PMC6368365; doi:10.1128/mBio.02662-18)
Supplement: FIG S3 [file mBio.02662-18-sf003.pdf]

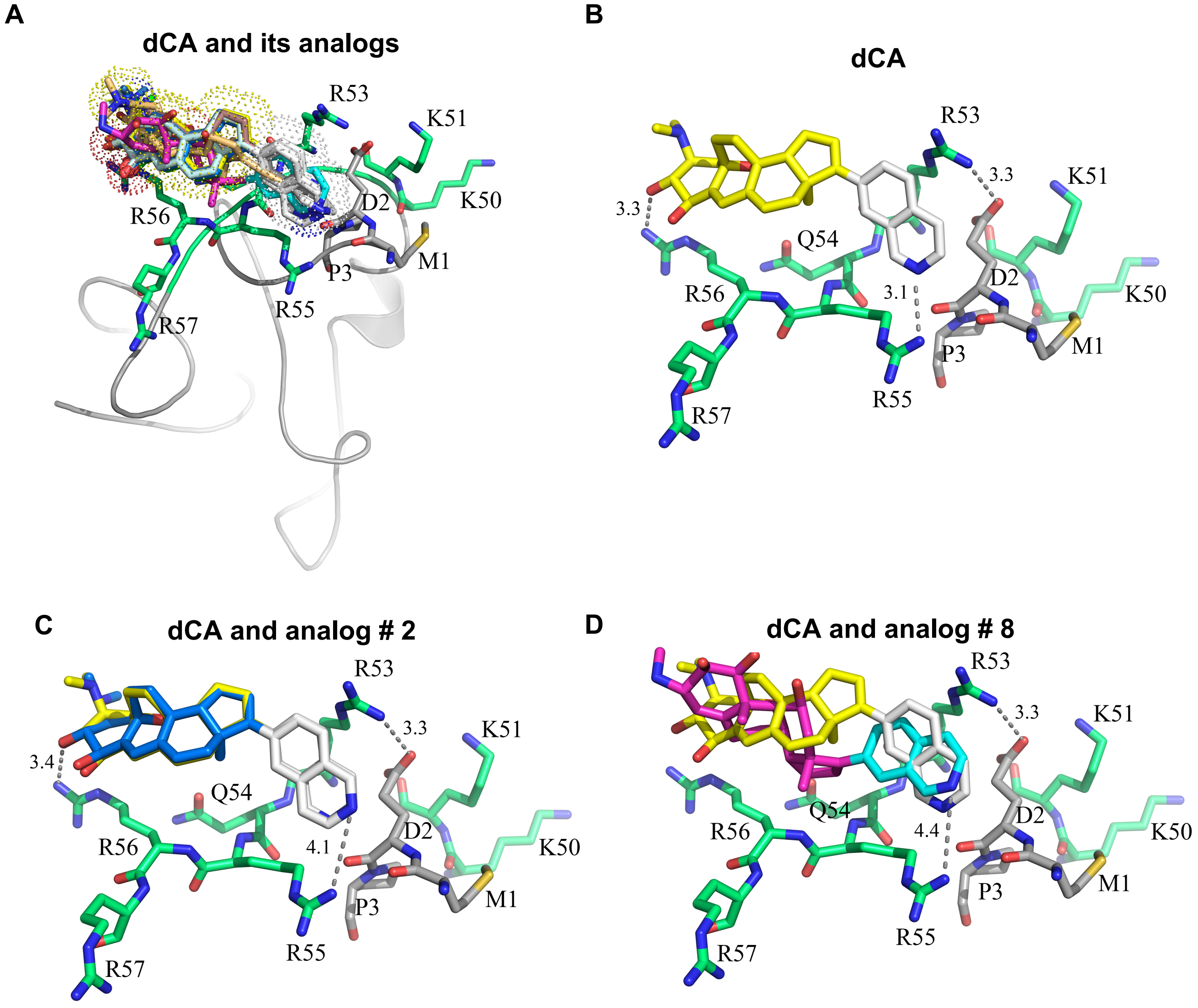

Figure S3. Molecular modeling of Tat PDB entry 1JFW interaction with dCA. (**A**) The small molecule dCA and other analogs with similar scaffold preferentially docked to the basic patch of HIV-1 Tat protein. The NMR structure PDB entry 1JFW of the HIV-1 Tat protein is used as a template for docking experiments. (**B**) Close-up view of the best pose of dCA molecule binding to Tat in the docking analysis. Basic patch residues of the NMR ensemble are shown in stick representation and the ligand dCA is shown in yellow color. (**C, D**) Close-up view of two inactive analogs of dCA (analog # 2 and # 8) binding site and the interacting residues in HIV-1 Tat protein.
