# Supplementary material for: Didehydro-Cortistatin A Inhibits HIV-1 by Specifically Binding to the Unstructured Basic Region of Tat
Source: mBio. 2019 Feb 5;10(1):e02662-18. doi: 10.1128/mBio.02662-18 (PMC6368365; doi:10.1128/mBio.02662-18)
Supplement: FIG S4 [file mBio.02662-18-sf004.pdf]

Figure S4. Mediouni *et al.*

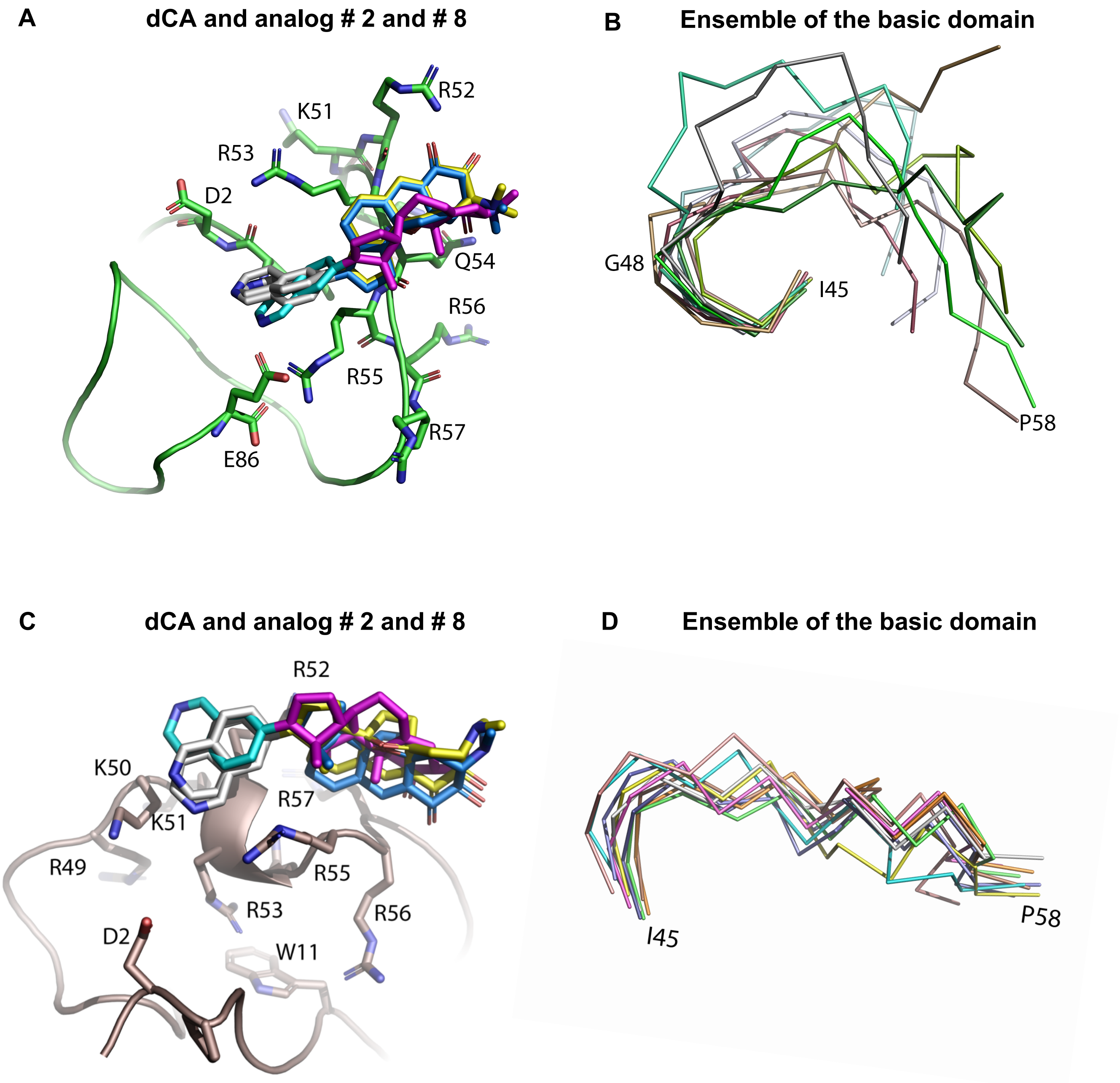

Figure S4. Molecular modeling of Tat PDB entry 1TIV and 1TBC binding to dCA. (**A**) dCA preferentially docked to the basic patch of HIV-1 Tat protein in model 1 of the structure PBD entry 1TIV. Of note, residue Arg[55], important for dCA binding to Tat, is buried under the C-terminus of the Tat-protein. (**B**) Ensemble of the conformations of the basic domain of PBD entry 1TIV, from residues ile[45] (I45) to pro[58] (P58), shown in stick representation. (**C**) Analysis of docking results using PBD entry 1TBC model as a template showed similar docking orientations as PBD entry 1K5K model. Of note, some of the basic residues are buried in this structure and arg[53] guanidinium group is in close proximity to tryptophan indole ring, which is energetically not favorable. Basic patch residues of the NMR ensemble are shown in stick representation and the ligand dCA is shown in yellow. Docking analysis of other inactive analogs, analog # 2 (in blue) and # 8 (in pink), are also shown for PBD entry 1TIV and 1TBC models. All docking experiments were performed as for the PBD entry 1K5K template. (**D**) Ensemble of the conformations of the basic domain of HIV-1 Tat in PBD entry 1TBC model, from residues ile[45] (I45) to pro[58] (P58), shown in stick representation.
