# Supplementary figures and images for: Didehydro-Cortistatin A Inhibits HIV-1 by Specifically Binding to the Unstructured Basic Region of Tat
Source: mBio. 2019 Feb 5;10(1):e02662-18. doi: 10.1128/mBio.02662-18 (PMC6368365; doi:10.1128/mBio.02662-18)

Figure S5. Mediouni *et al.*

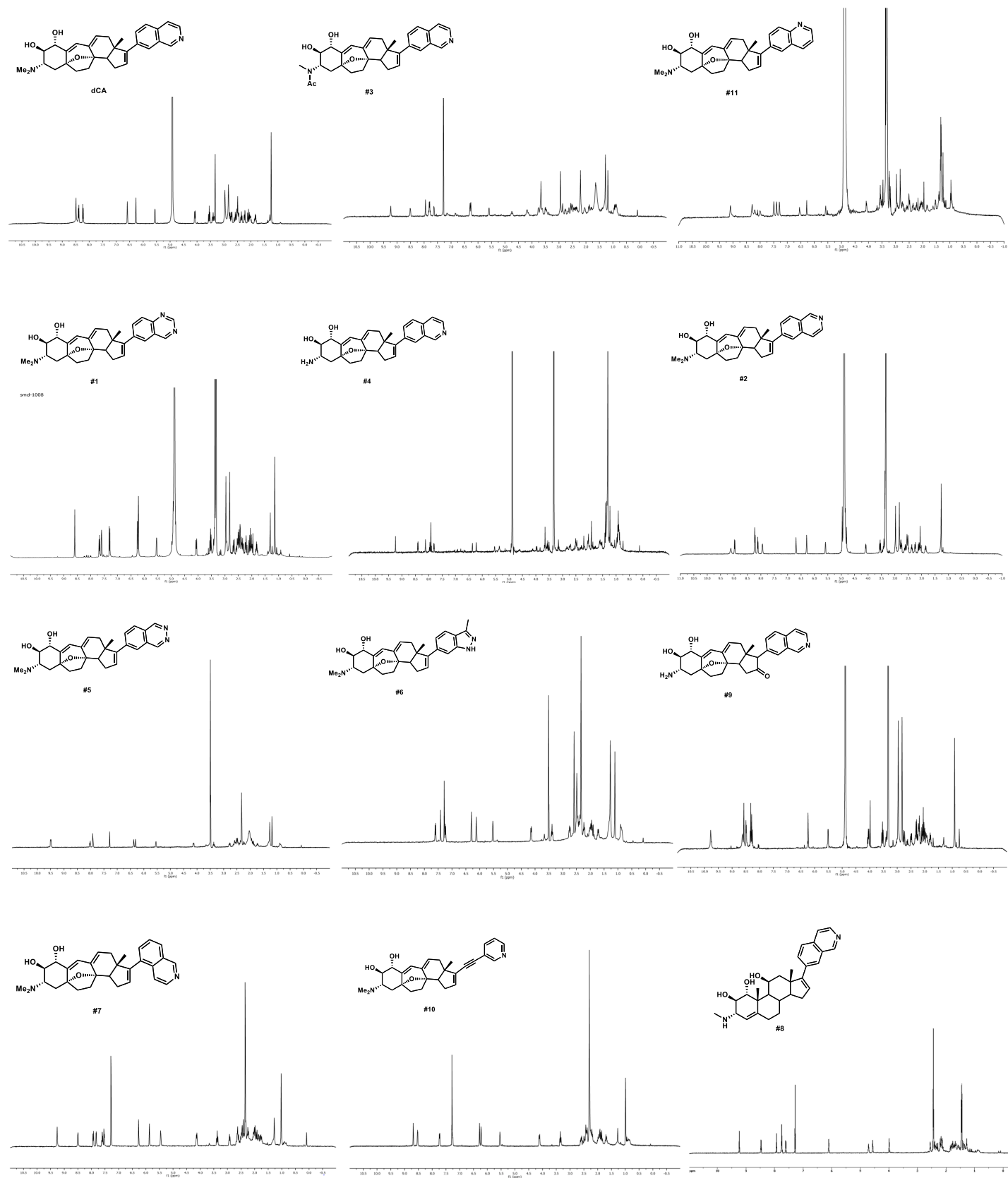

Figure S5. NMR profiles of dCA and analogs.

Supplement: FIG S5 [file mBio.02662-18-sf005.pdf]
