# Supplementary material for: Didehydro-Cortistatin A Inhibits HIV-1 by Specifically Binding to the Unstructured Basic Region of Tat
Source: mBio. 2019 Feb 5;10(1):e02662-18. doi: 10.1128/mBio.02662-18 (PMC6368365; doi:10.1128/mBio.02662-18)
Supplement: FIG S6 [file mBio.02662-18-sf006.pdf]

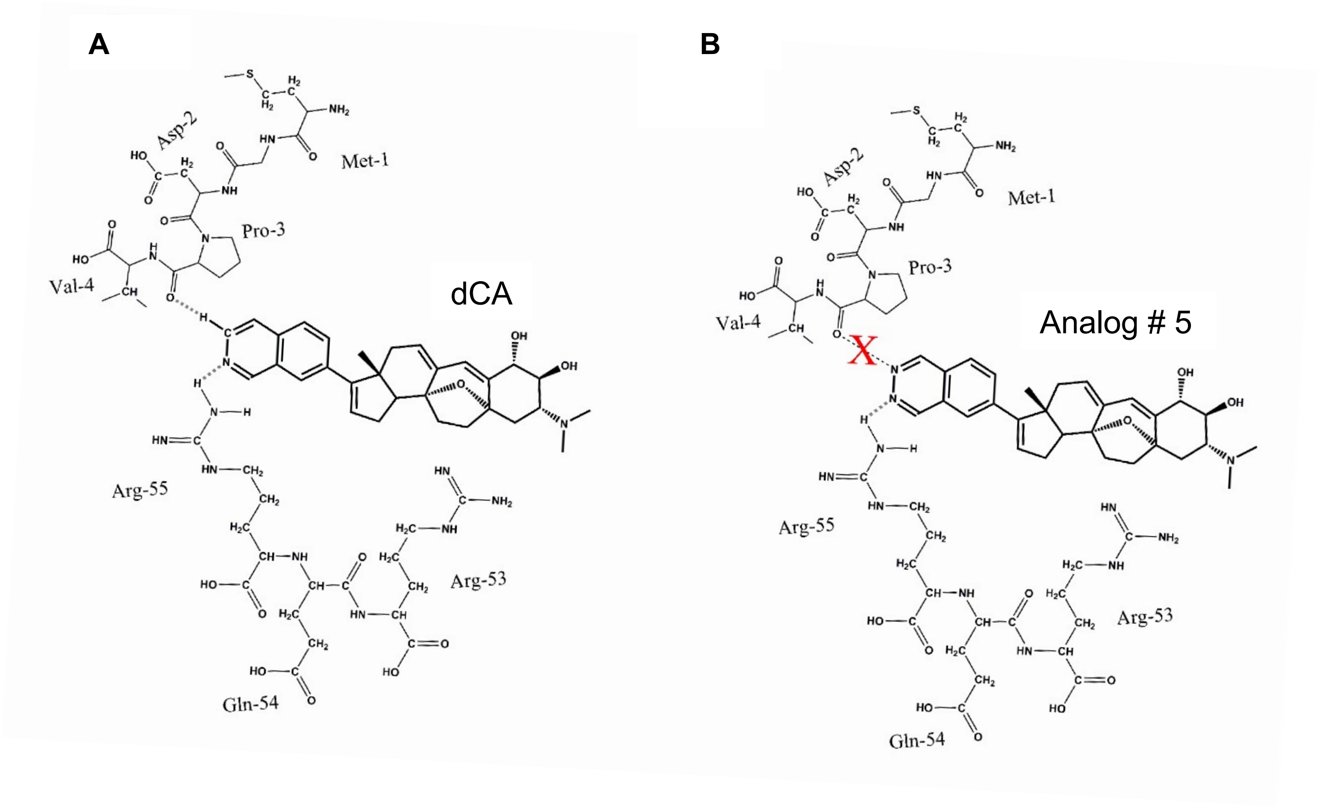

Figure S6. Structure-function activity of dCA and analog # 5. (**A**) dCA contains an isoquinoline heterocyclic group and the nitrogen atom of this group interacts with -NH2 moiety from the guanidinium group of Arg^{55} and the C-H group adjacent is in hydrogen bonding distance to the backbone carbonyl of Pro^{3} residue from the N-terminus of Tat. (**B**) Analog # 5 contains a phthalazine heterocyclic group with two adjacent nitrogens and one of the nitrogen atom of this group orients similar to dCA in our docking analysis and the adjacent nitrogen atom precludes the formation of a hydrogen bond with the backbone residues from the N-terminus of Tat.
