# Supplementary material for: Didehydro-Cortistatin A Inhibits HIV-1 by Specifically Binding to the Unstructured Basic Region of Tat
Source: mBio. 2019 Feb 5;10(1):e02662-18. doi: 10.1128/mBio.02662-18 (PMC6368365; doi:10.1128/mBio.02662-18)
Supplement: FIG S7 [file mBio.02662-18-sf007.pdf]

A

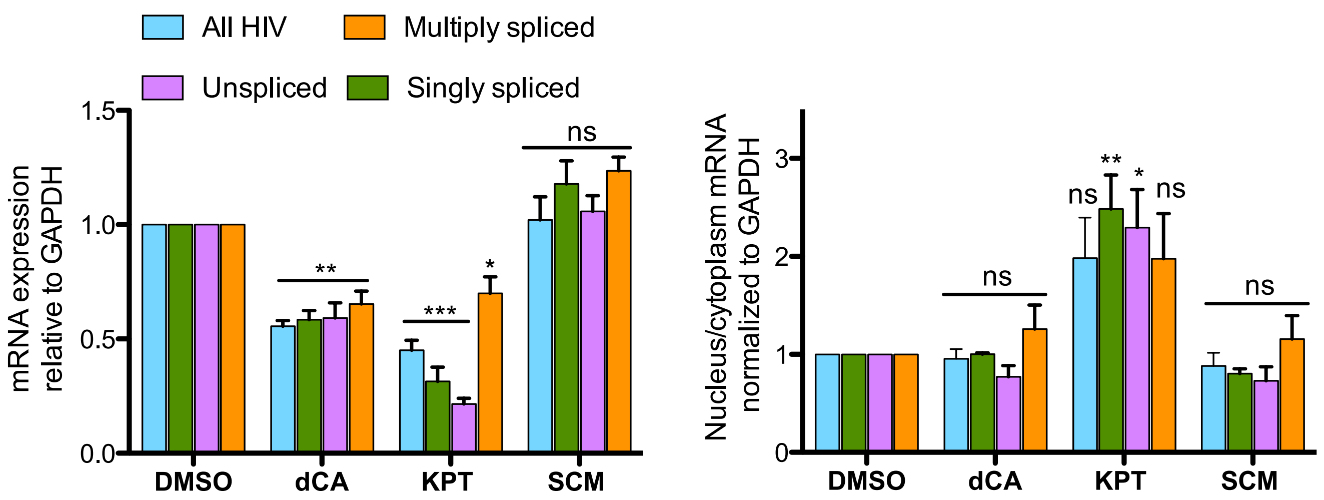

B

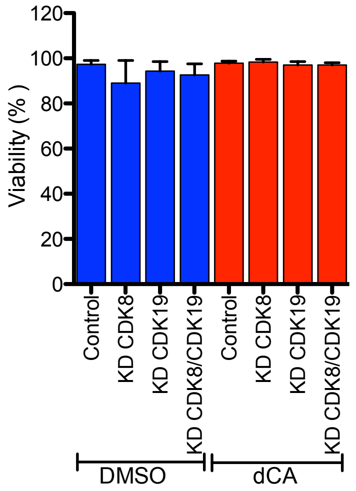

Figure S7. A. dCA does not perturb the export function of HIV Rev in CEM-SS cells. Cells were infected with NL4-3 strain for 24 hrs. The next day, compounds (dCA 30 nM, KPT 600 nM and SCM 30 nM) were added for 24 hrs. Total, nuclear and cytoplasmic mRNAs were extracted and viral messages measured by qRT-PCR. GAPDH was used for normalization. Data is a mean ± SEM of n=3 independent experiments. Statistical significance was determined using one way Anova with post-hoc Turkey test, comparing DMSO condition to the other conditions, $p < 0.0001$: ***, $p < 0.001$: **, $p < 0.01$: *. **B. Measure of the impact of CDK8 knockdown on HIV expression**. The anti-HIV activity of dCA, in acute infection of HeLa CD4 cells, is independent of CDK8. HeLa CD4 cells were transduced with shRNAs against CDK8 or CDK19 or both and selected with puromycin to stabilized shRNAs. Cells were then infected with NL4.3 strain for 24 hrs, in presence of DMSO or dCA (200 nM). After 24 hrs, cells were washed and fresh media with compounds was added. Seventy hrs later, viability was measured. Show is the mean ± SEM of n=3 independent experiments.
