# Supplementary material for: Didehydro-Cortistatin A Inhibits HIV-1 by Specifically Binding to the Unstructured Basic Region of Tat
Source: mBio. 2019 Feb 5;10(1):e02662-18. doi: 10.1128/mBio.02662-18 (PMC6368365; doi:10.1128/mBio.02662-18)
Supplement: FIG S8 [file mBio.02662-18-sf008.pdf]

A

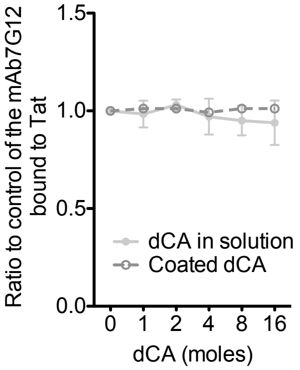

B

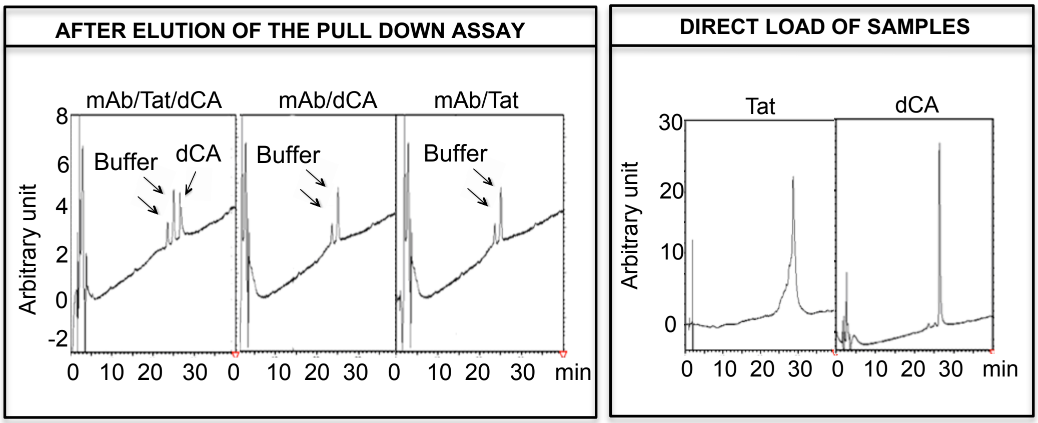

	Retention time (min)	Retention time in pull down assay (min)
Glycine HCL	23.44-24.96	
Tat	29	Under threshold of detection
dCA	26.64	
7G12	Not detected	

Figure S8. dCA recognizes folded Tat in solution. (**A**) dCA does not interfere with the interaction of Tat with mAb 7G12 by ELISA. mAb 7G12 is an antibody recognizing a tertiary structure of Tat. dCA was either pre-incubated with Tat and then the mixture was coated on a 96-well plate or dCA was added after blocking step (“dCA in solution”). Data is represented as the ratio of the signal of the mAb 7G12 bound to Tat in each condition relative to control signal without dCA. Data is a mean ± SD of n=2 independent experiments. **(B**) dCA recognizes folded Tat after pulled down with mAb7G12. The mAb 7G12 was used at equimolar concentration with Tat. dCA was then added to the mix and HPLC was used to reveal the interaction Tat-dCA. Data is representative of n=3 independent experiments.
