# Supplementary material for: Didehydro-Cortistatin A Inhibits HIV-1 by Specifically Binding to the Unstructured Basic Region of Tat
Source: mBio. 2019 Feb 5;10(1):e02662-18. doi: 10.1128/mBio.02662-18 (PMC6368365; doi:10.1128/mBio.02662-18)
Supplement: FIG S9 [file mBio.02662-18-sf009.pdf]

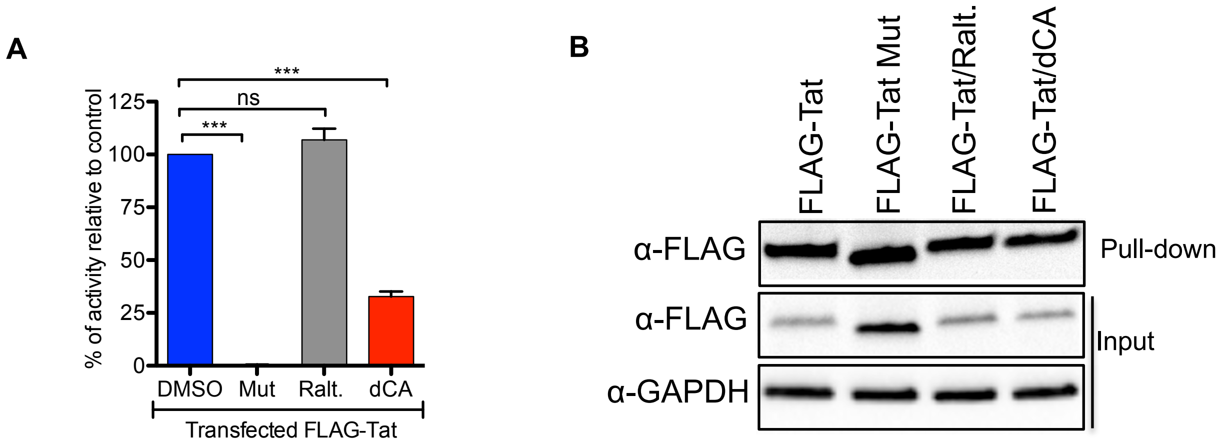

Figure S9. Tat-TAR interaction blocked by dCA in HeLa-CD4-LTR-Luc cells. (**A**) Transactivation assays. HeLa-CD4-LTR-Luc cells, transfected with Tat or Tat Mut, were incubated with the indicated compounds (dCA, Raltegravir "Ralt.", 25 nM) or DMSO. Forty-eight h later, cells were lysed and cross-linked and luciferase activity relative to Bradford assay or ChIP TAR RNA (**Figure 6D**) were performed. Data is the mean ± SD of n=3 independent experiments. (**B**) (Top) Western blot of the amount of FLAG-Tat bound to FLAG-beads after elution of the samples for ChIP TAR RNA, as control input; (bottom) the input of FLAG-Tat and GAPDH as loading control. Data is representative of n=3 independent experiments. Statistical significance was determined using one way Anova with post-hoc Turkey test, comparing DMSO condition to the other conditions, $p < 0.0001$: ***.
