# Supplementary material for: Didehydro-Cortistatin A Inhibits HIV-1 by Specifically Binding to the Unstructured Basic Region of Tat
Source: mBio. 2019 Feb 5;10(1):e02662-18. doi: 10.1128/mBio.02662-18 (PMC6368365; doi:10.1128/mBio.02662-18)
Supplement: TEXT S1 [file mBio.02662-18-s0001.docx]

**SUPPLEMENTAL METHODS**

**Bacterial expression and purification of Tat.** It was performed as previously described (13). Briefly, pTatC6H-1 (NIH AIDS Reagent Program) plasmid encoding C-terminal 6xHistidine Tat_1-86_ (HXB3, subtype B) was expressed in E. coli BL 21 to obtain Tat protein. This plasmid was also point mutated in the basic domain ^49^RKK**RR**Q**RRR**PP^59^ (arginine residues mutated to alanine residues) to obtain Tat Mut. The induction of the proteins was performed with isopropyl β-D-thio-galactopyranoside (IPTG) and purification done as recommended in the AIDS Reagent Program protocol, with an added second purification step using a 30 kDa filter (Millipore). Purified proteins were then solubilized in acidic phosphate buffer to preserve cysteine residues from oxidation.

**Tat-dCA co-immunoprecipitation followed by HPLC**. Protein G coupled agarose beads were incubated with mAb 7G12, recognizing a conformational epitope on Tat previously described (48), overnight at 4°C. Then, Tat (50 µg) were added to the beads for 2 hr at RT, followed by 3.12 µg of dCA for 1 hr at RT. After each step, beads were washed with PBS. The complex mAb 7G12-Tat-dCA was removed from beads by incubating with glycine HCL (Sigma). The supernatant was analysed by HPLC with a C8 reverse phase column using buffer A (water supplemented with 0.1 % (v/v) trifluoroacetic acid (Sigma)) and buffer B (acetonitrile (Merck) supplemented with 0.1 % (v/v) trifluoroacetic acid). Gradient was buffer B from 10-50 % in 40 min with a 0.8 mL/min flow rate.

**Detection of Tat-mAb 7G12 interaction in presence of dCA**. Tat alone or Tat and dCA (0 to 16 moles) (called “mix Tat”) were coated onto a 96-well ELISA plate. dCA or control buffer were added for 1 hr at RT. Then, mAb 7G12 was added at equimolar concentration with Tat for 1 hr at RT, followed by HRP-conjugated anti-mouse antibody (GE Healthcare) for 1 hr at RT. 2.2′-azino-di (3-ethylbenzothiazoline-6-sulfonate) (ABTS) substrate was added. The absorbance was measured at a wavelength of 405 nm 1 hr later. Washes were performed after each step.

**Isothermal titration calorimetry**. The measurements were conducted at 25˚C on a Microcal VP-ITC (Microcal, Inc.; Northampton MA). Titrations of HIV Tat HXB3 and HIV-1 TAR with dCA (or peptide, ^46^SYGRKKRRQRRRPPQ^60^) were done by injecting 250 μL into an isothermal sample chamber containing 1.5 mL HIV Tat HXB3 or HIV-1 TAR solution that was 10 μM in strand. Each experiment of this type was accompanied by the corresponding control experiment in which peptide or dCA injected into a solution of buffer alone. The duration of each injection was 8 s and the delay between the injections was 300 s and 180 s, the initial delay prior to the first injection was 300 s. Each injection generated a heat burst curve (microcalories/second vs. seconds) and the area under each curve was determined by integration [using the origin version 7.0 software (Microcal, Inc.; Northampton, MA)] to obtain the measure of the heat associated with that injection.

**Chromatin immunoprecipitation assay (CHIP).** HeLa-CD4-LTR-Luc cells (4×10^6^) were transfected with different constructs (PGK-FLAG Tat and PGK-FLAG Tat Mut). Twenty-four hours later, cells were plated at 4×10^6^ cells into a 10 cm^2^ dish in presence of compounds for 48 hrs. Cells were then split and half used to determine luciferase activity and the other half cross-linked with 1 % formaldehyde to perform CHIP assay. Pellets of 1×10^7^ cells were sonicated 12 times for 10-s bursts on ice to generate sheared chromatin of 200 to 500 nucleotides. The equivalent of 8.5×10^6^ cells was used for each immunoprecipitation assay with magnetic FLAG beads M2 (Sigma). Chromatin was saved as an input control. Immunoprecipitated DNA was eluted with 500 μL of elution buffer (1 % Dodecyl sulfate, 100 mM sodium bicarbonate). The reverse cross-link was performed by adding 20 μL of 5 M NaCl and incubation for 4 h at 65°C. Digestion of contaminating proteins was then carried out by adding 10 μL of EDTA 0.5 M, 20 μL of Tris HCl pH 6.5, 1 M and 2 μL of 2 mg/mL of proteinase K. DNA was purified by using PCR Clean (Promega). Primers used were TAR forward (AGCTTTCTACAAGG-GACTTTCCGC) and TAR reverse (ATTGAGG-CTTAAGCAGTGGGTTCC) (62). Input was used to standardize the values obtained. The relative proportions of co-immunoprecipitated DNA were determined on the basis of the threshold cycle for qRT-PCR. Western blots were performed with an anti- FLAG (1:2000, Sigma), anti-GAPDH (1:500, Santa Cruz) and anti-Mouse Trueblot (1:2000, Rockland).

**RNA immunoprecipitation of 7SK snRNP**. The immunoprecipitation of the 7SK snRNP by intracellular Hexim-1 was performed as previously described in ([42](#_ENREF_42)) with some modifications. Briefly, HeLa-CD4-LTR-LacZ cells were incubated with testing compounds. After 48 hrs, cells were lysed with buffer A (20 mM HEPES-KOH pH 7.8, 0.2 mM EDTA, 0.5 % Nonidet P-40, 100 mM KCl) supplemented with protease inhibitor, on ice for 10 min. Cell lysates were centrifuged at 5000 × g for 5 min at 4°C, and supernatants were collected. Supernatants were then pre-cleared with protein A/G+ beads and divided into three aliquots. Each aliquot supplemented with RNAse inhibitors was incubated with 1 μg of normal rabbit IgG or anti-Hexim antibody overnight at 4°C or kept for input. Samples were then incubated with 30 μL of protein A/G+ beads pre-coated with Bovine serum albumin and yeast tRNA for an additional 2 h 30 at 4°C. Beads were washed five times with buffer A. RNA was then extracted by Trizol (Invitrogen), reverse transcribed and analyzed by qRT-PCR. Data were normalized to input amounts of 7SK snRNA. For the RNAse treatment conditions, samples were incubated with RNA A enzyme (Epicentre) for 20 min at 37°C.

**Transactivation assay. In HeLa-CD4-LTR-Luc cells and OM10.1 cells.** The activity of Tat was verified by a transactivation assay as previously reported (13). Briefly, HeLa-CD4-LTR-Luc cells and OM10.1 were plated at 1×10^5^ and 8x10^5^ cells per well of a 6-well plate respectively. The following day, recombinant Tat protein was added to the cells in culture media without serum and in the presence of 100 μM of Chloroquine. Five hours later, serum was added at 5 % to the culture media. Cells were lysed 24 hrs later, protein concentration determined by Bradford assay and luciferase activity was measured for HeLa-CD4-LTR-Luc cells. Results corresponding to luciferase activity per protein concentration of each sample are shown as relative light unit (RLU). As for the OM10.1 cells, RNA was extracted using the RNA extraction kit (Qiagen) and DNA contamination was removed using Turbo-DNAse kit (Ambion). First-strand cDNA was synthesized using SuperscriptIII^TM^ first-strand cDNA kit (Invitrogen) and random hexamers as first-strand primers. qRT-PCR was performed with an aliquot of cDNA as template, using LightCycler® 480 SYBR Green I Master in a 20 µL reaction according to the manufacturer’s instructions. The mRNAs from each sample were used as negative control. Primer sequences for GAPDH (13) and all viral mRNAs as previously described (59). **In HeLa-CD4-LTR-LacZ cells**. It was performed as previously described (10). Briefly, cells were plated at 1×10^4^ cells per well of a 96-well plate. Twenty-four hrs later HIV-1 NL4-3 strain was added in the presence of testing compound or DMSO. Seventy-two hrs post infection, cells were disrupted in lysis buffer (60 mM Na_2_HPO_4_, 40 mM NaH_2_PO_4_, 10 mM KCl, 10 mM MgSO_4_, 2.5 mM EDTA, 50 mM ß-mercaptoethanol, 0.125 % Nonidet P-40) and a quantitative chlorophenol red-ß-D-galactopyranoside (CPRG)-based (Boehringer Mannheim) assay was performed. The cell extracts were incubated in a reaction buffer (0.9 M phosphate buffer [pH 7.4], 9 mM MgCl_2_, 11 mM ß-mercaptoethanol, 7 mM CPRG) until a red color developed and measured with an LP400 (Becton Dickinson) plate reader at 572 nm.

**Half-life of Tat *in vivo*.** HeLa-CD4-LTR-Luc cells were plated at 3×10^5^ cells per well in a 24-well plate. The next day, Tat was added to the cells in serum free media for 4 hrs. Cells were washed with phosphate buffer saline (PBS) and compounds were added in complete media for an additional 6 hrs. Cells were washed and lysed with RIPA buffer supplemented with protease inhibitors. Total protein concentration was assessed by Bradford assay and Tat protein in each condition determined by western blot with an anti-His antibody at 1:1500 dilution (Thermofisher). An anti-actin antibody at 1:5000 dilution (Thermofisher) was used as control. Results represent Tat bands normalized to actin bands. The proteasome inhibitor MG132 (10 μM, Sigma) was used as control.

**EMSA With full-length Tat:** similar protocol was performed as in Das *et al*. (60)**. With Tat’s basic peptide:** the 59-nucleotides TAR RNA was prepared by *in vitro* T7 RNA polymerase transcription according to Maxiscript kit manufacturer’s instructions (Ambion). The radiolabeled RNA was purified by urea gel followed by phenol-chloroform extraction. Binding reaction mixture (25 µL of 10 mM Tris HCL pH 7.5, NaCl 70 mM, EDTA 0.2 mM, NP40 0.01 %, Glycerol 5 % and tRNA 100 ng/µL) contained traces of radiolabeled TAR RNA and basic peptide (Pep B, residues 45-59) (or peptide C “Pep C”, residues 69-83, as negative control) in combination or not with compounds or cold TAR. The mixture was incubated for 10 min at room temperature (RT). Bromophenol powder was added and 20 µL of each sample was loaded on a native polyacrylamide gel at 200 V for 1 hr. The gel was dried and bands revealed with a phosphor-imager.

**dCA and analogs synthesis.**

**dCA:**

Compound A was dissolved in DMSO (300 L, 0.06 M). To this solution was added 7-trimethylstannylisoquinoline (20 mg, 68 μmοl, 4 equiv.), CuCl (15 mg, 170 μmοl, 10 equiv.), LiCl (7 mg, 170 μmοl, 10 equiv.) and Pd(PPh_3_)_4_ (10 mg, 85 μmοl, 0.5 equiv.). The mixture was degassed by bubbling argon through the solution for 10 min and then immersed in a preheated oil bath at 60°C for 1 hr. The resulting mixture was allowed to cool to RT, diluted with EtOAc (5 mL) and washed with 5 % aq. NH_4_OH. The layers were partitioned and the aqueous layer was extracted four times (4 x 5 mL) with EtOAc. The combined organic portions were washed with sat. aq. NaCl (5 mL), dried over Na_2_SO_4_, filtered, and concentrated in vacuo. The isolated residue was purified by PTLC (NH_3_ deactivation; 10 % MeOH:CH_2_CI_2_) to obtain dCA (4 mg, 42 %) as a yellow foam. The 1H-NMR (CD_3_OD, 400 MHz) and MS (ESI) at m/z All [M+H]^+^ of dCA were obtained for characterization.

**#1**

Compound A (10 mg, 21 μmοl), 6-trimethylstannylquinazoline (7.5 mg, 25 μmοl), Pd(PPh_3_)_4_ (12 mg, 10.5 μmοl), CuCl (21 mg, 210 μmοl) and LiCl (8.8 mg, 210 μmοl) were dissolved in degassed DMSO (0.35 ml), then heated at 60 °C for 1 hr. The mixture was allowed to cool to RT, diluted with DCM (5 mL) and washed with 5 % aq. NH_4_OH. The layers were partitioned and the aqueous layer was extracted with DCM (4 x 5 mL). The combined organic portions were washed with sat. aq. NaCl (5 mL), dried over Na_2_SO_4_, filtered, and concentrated in vacuo. The crude mixture was purified by HPLC to obtain compound #1 (3.2 mg, 23 %) as a yellow foam. The 1H-NMR (CD_3_OD, 400 MHz) and MS (ESI) m/z 472 [M+H] ^+^ of compound #1 were obtained for characterization.

**#2**

Compound A (10 mg, 21 μmοl), 7-trimethylstannylisoquinoline (7.3 mg, 25 μmοl), Pd(PPh_3_)_4_ (12 mg, 10.5 μmοl), CuCl (21 mg, 210 μmοl) and LiCl (8.8 mg, 210 μmol) were dissolved in degassed DMSO (0.35 mL), then heated at 60 °C for 1 hr. The mixture was allowed to cool to RT, diluted with DCM (5 mL) and washed with 5 % aq. NH_4_OH. The layers were partitioned and the aqueous layer was extracted with DCM (4 x 5 mL). The combined organic portions were washed with sat. aq. NaCl (5 mL), dried over Na_2_SO_4_, filtered, and concentrated in vacuo. The crude mixture was purified by HPLC to obtain compound #2 (1.7 mg, 12 %) as a yellow foam. The 1H-NMR (CD_3_OD, 400 MHz) and MS (ESI) m/z 471 [M+H] ^+^ of compound #2 were obtained for characterization.

**#3**

To a solution of compound 4a (1.1 mg, 2.4 μmοl) in water (0.1 mL) and 2N HC1 (0.1 mL) was added Ac_2_O (68 mg, 7.2 μmοl), NaHCO_3_ (2.0 mg, 24 μmοl) at 25 °C and DCM (0.2 ml), the resulting mixture was then stirred for 1 hr. The layers were partitioned and the aqueous layer was extracted with DCM (4 x 5 mL). The combined organic portions were dried over Na_2_SO_4_, filtered, and concentrated in vacuo. The crude mixture was purified by PTLC to obtain compound #3 (1.0 mg, 83 %) as a yellow foam. The 1H-NMR (CDC1_3_, 400 MHz) and MS (ESI) m/z 499 [M+Hf of compound #3 were obtained for characterization.

**#4**

To a solution of dedihydro-cortistatin A (4.7 mg, 10 μηιοΐ) in MeOH (0.1 mL) and H_2_O (0.02 mL) was added NaOAc (8.2 mg, 100 μmοl) and iodine (3.8 mg, 15 μmοl), the resulting mixture was then stirred for 1 hr at 45 °C. Subsequently, the mixture was treated with 2N NaOH solution (0.1 mL) and iodine (2.5 mg, 10 μmοl) at 45 °C and stirred for 1 hr. The mixture was diluted with DCM and washed with NaS_2_O_3_ solution and NaHCO_3_. The layers were partitioned and the aqueous layer was extracted with DCM (4 x 5 mL). The combined organic portions were washed with sat. aq. NaCl (5 mL), dried over Na_2_SO_4_, filtered, and concentrated in vacuo. The crude mixture was purified by PTLC to furnish compound #4 (3.6 mg, 77 %) and compound Z (1.1 mg, 23 %). The 1H-NMR (CDC1_3_, 400 MHz) and MS (ESI) m/z 457 [M+H]^+^ of compound #4 and the 1H-NMR (CD_3_OD, 400 MHz) and MS (ESI) m/z 443 [M+H]^+^ of compound #4 were obtained for characterization.

**#5**

Compound A (9.4 mg, 20 μmοl), 6-trimethylstannylphthalazine (11.7 mg, 40 μmοl, Pd(PPh_3_)_4_ (11.6 mg, 10 μmοl), CuCl (19.8 mg, 200 μmοl) and LiCl (10.2 mg, 240 μmοl) were dissolved in degassed DMSO (0.8 ml), then heated at 60 °C for 1 hr. The mixture was allowed to cool to RT, diluted with DCM (5 mL) and washed with 5 % aq. NH_4_OH. The layers were partitioned and the aqueous layer was extracted with DCM (4 x 5 mL). The combined organic portions were washed with sat. aq. NaCl (5 mL), dried over Na_2_SO_4_, filtered, and concentrated in vacuum. The crude mixture was purified by PTLC to furnish compound #5 (1.5 mg, 16 %) as a yellow foam. The 1H-NMR (CDCl_3_, 400 MHz) and MS (ESI) m/z 472 [M+H] ^+^of compound #5 were obtained for characterization.

**#6**

Compound A (9.4 mg, 20 μmοl), 3-methyl-6-trimethylstannyl-lH- indazole (11.7 mg, 40 μmοl), Pd(PPh_3_)_4_ (11.6 mg, 10 μmοl), CuCl (19.8 mg, 200 μmοl) and LiCl (10.2 mg, 240 μπιοΐ) were dissolved in degassed DMSO (0.8 ml), then heated at 60 °C for 1 hr. The mixture was allowed to cool to RT, diluted with DCM (5 mL) and washed with 5 % aq. ΝΗ_4_ΟΗ. The layers were partitioned and the aqueous layer was extracted with DCM (4 x 5 mL). The combined organic portions were washed with sat. aq. NaCl (5 mL), dried over Na_2_SO_4_, filtered, and concentrated in vacuo. The crude mixture was purified by PTLC to obtain compound #6 (1.2 mg, 12 %) as a yellow foam. The 1H-NMR (CDC1_3_, 400 MHz) and MS (ESI) m/z 474 [M+H] ^+^ of compound #6 were obtained for characterization.

**#7**

Compound A (9.4 mg, 20 μmοl), 5-trimethylstannylisoquinoline (9.7 mg, 40 μmοl), Pd(PPh_3_)_4_ (11.6 mg, 10 μmοl), CuCl (19.8 mg, 200 μmοl) and LiCl (10.2 mg, 240 μmοl) were dissolved in degassed DMSO (0.8 ml), then heated at 60 °C for 1 hr. The mixture was allowed to cool to RT, diluted with DCM (5 mL) and washed with 5 % aq. NH_4_OH. The layers were partitioned and the aqueous layer was extracted with DCM (4 x 5 mL). The combined organic portions were washed with sat. aq. NaCl (5 mL), dried over Na_2_SO_4_, filtered, and concentrated in vacuo. The crude mixture was purified by PTLC to furnish compound #7 (1.7 mg, 18 %) as a yellow foam. The 1H-NMR (CDC1_3_, 400 MHz) and MS (ESI) m/z 471 [M+H] ^+^of compound #7 were obtained for characterization.

**#8**

Compound 8a (18.4 mg, 40 μmοl), 7-trimethylstannylisoquinoline (23 mg, 80 μmοl), Pd(PPh_3_)_4_ (14 mg, 12 μmοl), CuCl (49 mg, 500 μmοl) and LiCl (20 mg, 500 μmοl) were dissolved in degassed DMSO (1 ml), then heated at 60 °C for 1 hr. The mixture was allowed to cool to RT, diluted with DCM (5 mL) and washed with 5 % aq. NH_4_OH. The layers were partitioned and the aqueous layer was extracted with DCM (4 x 5 mL). The combined organic portions were washed with sat. aq. NaCl (5 mL), dried over Na_2_SO_4_, filtered, and concentrated in vacuo. The crude mixture was purified by HPLC to furnish compound #8 (12 mg, 40 %) as a yellow foam. The 1H-NMR (CDCl_3_, 400 MHz) and MS (ESI) m/z 461 [M+H] ^+^of compound #8 were obtained for characterization.

**#9**

TFA salt of dedihydro-cortistatin A (160 mg) was left at -80 °C under air in a vial for 4 weeks. About 10 %) of dedihydro-cortistatin A was slowly oxidized and formed compound #9. The crude mixture was purified by HPLC to yield about 15 mg of compound #9 as pure compound. The 1H-NMR (CD_3_OD, 400 MHz) and MS (ESI) m/z 487 [M+H] ^+^ of compound #9 were obtained for characterization.

**#10**

Compound A (14 mg, 30 μmοl), 3-ethynylpyridine (15 mg, 150 μmοl), Pd(PPh_3_)_2_Cl_2_ (4.2 mg, 60 μmοl) Cul (1.1 mg, 60 μmοl), and Et_3_N (30 μί) were dissolved in degassed THF (0.30 ml), then heated at 60 °C for 2 hrs. The mixture was allowed to cool to RT, diluted with DCM (5 mL) and washed with 5 % aq. NH_4_OH. The layers were partitioned and the aqueous layer was extracted with DCM (4 x 5 mL). The combined organic portions were washed with sat. aq. NaCl (5 mL), dried over Na_2_SO_4_, filtered, and concentrated in vacuo, the crude mixture was purified by PTLC to obtain compound #10 (11.2 mg, 84 %) as a yellow foam. The 1H-NMR (CDC1_3_, 400 MHz) and MS (ESI) m/z 445 [M+H] ^+^ of compound #10 were obtained for characterization.

**#11**

Compound A (10 mg, 21 μmοl), quinolin-7-yl boronic acid (7.2 mg, 42 μmοl), Pd_2_(dba)_3_ (3.8 mg, 4.2 μmοl), Ruphos (6.9 mg, 16.8 μmοl) and potassium phosphate (13.3 mg, 63 μmοl) were dissolved in degassed n-BuOH (1 mL) and water (0.2 mL), then heated at 60 °C for 36 hrs. The mixture was allowed to cool to RT, diluted with EtOAc (5 mL) and washed with 5 % aq. NaHC0_3_. The layers were partitioned and the aqueous layer was extracted with EtOAc (4 x 5 mL). The combined organic portions were washed with sat. aq. NaCl (5 mL), dried over Na_2_SO_4_, filtered, and concentrated in vacuo. The crude mixture was purified by HPLC to obtain compound #11 (3.1 mg, 23 %) as a yellow foam. The 1H-NMR (CD_3_OD, 400 MHz) and MS (ESI) m/z 471 [M+H] ^+^ of compound #11 were obtained for characterization.
